# Phase II Trial of Concurrent Sunitinib and Image-Guided Radiotherapy for Oligometastases

**DOI:** 10.1371/journal.pone.0036979

**Published:** 2012-06-27

**Authors:** Charles C. L. Tong, Eric C. Ko, Max W. Sung, Jamie A. Cesaretti, Richard G. Stock, Stuart H. Packer, Kevin Forsythe, Eric M. Genden, Myron Schwartz, K. H. Vincent Lau, Matthew Galsky, Junko Ozao-Choy, Shu-hsia Chen, Johnny Kao

**Affiliations:** 1 Department of Radiation Oncology, Mount Sinai Medical Center, New York, New York, United States of America; 2 Department of Otolaryngology-Head and Neck Surgery, Mount Sinai Medical Center, New York, New York, United States of America; 3 Department of Medical Oncology, Mount Sinai Medical Center, New York, New York, United States of America; 4 Florida Radiation Oncology Group, Jacksonville, Florida, United States of America; 5 Surgical Oncology, Mount Sinai Medical Center, New York, New York, United States of America; 6 Department of Oncological Sciences, Mount Sinai Medical Center, New York, New York, United States of America; 7 Department of Radiation Oncology, Good Samaritan Hospital Medical Center, West Islip, New York, United States of America; The University of Chicago, United States of America

## Abstract

**Background:**

Preclinical data suggest that sunitinib enhances the efficacy of radiotherapy. We tested the combination of sunitinib and hypofractionated image-guided radiotherapy (IGRT) in a cohort of patients with historically incurable distant metastases.

**Methods:**

Twenty five patients with oligometastases, defined as 1–5 sites of active disease on whole body imaging, were enrolled in a phase II trial from 2/08 to 9/10. The most common tumor types treated were head and neck, liver, lung, kidney and prostate cancers. Patients were treated with the recommended phase II dose of 37.5 mg daily sunitinib (days 1–28) and IGRT 50 Gy (days 8–12 and 15–19). Maintenance sunitinib was used in 33% of patients. Median follow up was 17.5 months (range, 0.7 to 37.4 months).

**Results:**

The 18-month local control, distant control, progression-free survival (PFS) and overall survival (OS) were 75%, 52%, 56% and 71%, respectively. At last follow-up, 11 (44%) patients were alive without evidence of disease, 7 (28%) were alive with distant metastases, 3 (12%) were dead from distant metastases, 3 (12%) were dead from comorbid illness, and 1 (4%) was dead from treatment-related toxicities. The incidence of acute grade ≥ 3 toxicities was 28%, most commonly myelosuppression, bleeding and abnormal liver function tests.

**Conclusions:**

Concurrent sunitinib and IGRT achieves major clinical responses in a subset of patients with oligometastases.

**Trial Registration:**

ClinicalTrials.gov NCT00463060

## Introduction

The standard non-surgical approach to distant metastases from solid tumors is systemic therapy alone with radiation therapy reserved for palliation of local symptoms [1]. In the setting of oligometastases, defined as metastatic deposits that are limited in number and location, incorporating local therapy is a conceptually attractive approach [2]. Five recently published clinical trials demonstrated high rates of local control for lung, liver, bone, adrenal, soft tissue and lymph node metastases treated with intensive radiation dose-fractionation schedules using image-guided stereotactic radiotherapy [3]–[7]. The rationale for administering curative-intent radiation for oligometastases is that a proportion of these patients will have durable remissions with an acceptable toxicity profile [1]. In these studies, approximately 20% of patients remained free of recurrence several years after treatment when all sites of disease can be targeted by radiation [7], [8]. However, most patients develop additional distant metastases within months of treatment [4]–[7]. These data highlight the need for effective systemic agents for the majority of patients. In turn, clinical models suggest that the relative importance of effective local therapy increases as systemic therapy becomes more effective [9].

Testing the hypothesis that distant metastases can be delayed or prevented by systemic therapy requires an approach similar to adjuvant treatment of primary cancers. In the studies recently reported by Rusthoven et al. and Lee et al., chemotherapy was discontinued for at least 4 weeks before, during and after stereotactic body radiation to sites of metastatic involvement [3], [5], [6]. In contrast, concurrent systemic therapy offers radiosensitization and simultaneously addresses the competing risks of local and distant progression [10]. Choosing a rational systemic agent for the heterogeneous population of oligometastasis is a challenge. With the hypothesis that targeting angiogenesis and tumor-mediated immune suppression represents an important target in most types of cancer, we identified sunitinib, a multitargeted tyrosine kinase inhibitor of VEGFR1, VEGFR2, VEGFR3, PDGFR, c-kit, FLT3 and ret, as a potential enhancer of response to radiotherapy [11], [12]. In addition to effects on angiogenesis, our group demonstrated robust effects of sunitinib on immunosuppressive myeloid-derived suppressor cells (MDSC) [13]. MDSC and T regulatory cells (Treg) are important mediators in immune suppression. In our preclinical model, treatment with sunitinib decreased the number of MDSC and Treg in tumor-bearing mice [13]. We have previously reported phase I results from a clinical trial of concurrent sunitinib and hypofractionated image-guided radiation therapy for patients of oligometastases with a 1-year progression-free survival of 44% [14]. We now report results of a phase II trial investigating concurrent sunitinib and image-guided radiation therapy for patients with oligometastases.

## Methods

### Ethics Statement

The study (NCT00463060) was approved by the Mount Sinai School of Medicine institutional review board, and was conducted in accordance with federal and institutional guidelines. All patients signed written informed consent. The protocol for this trial and supporting CONSORT checklist are available as supporting information; see Checklist S1 and Protocol S1.

**Figure 1:**
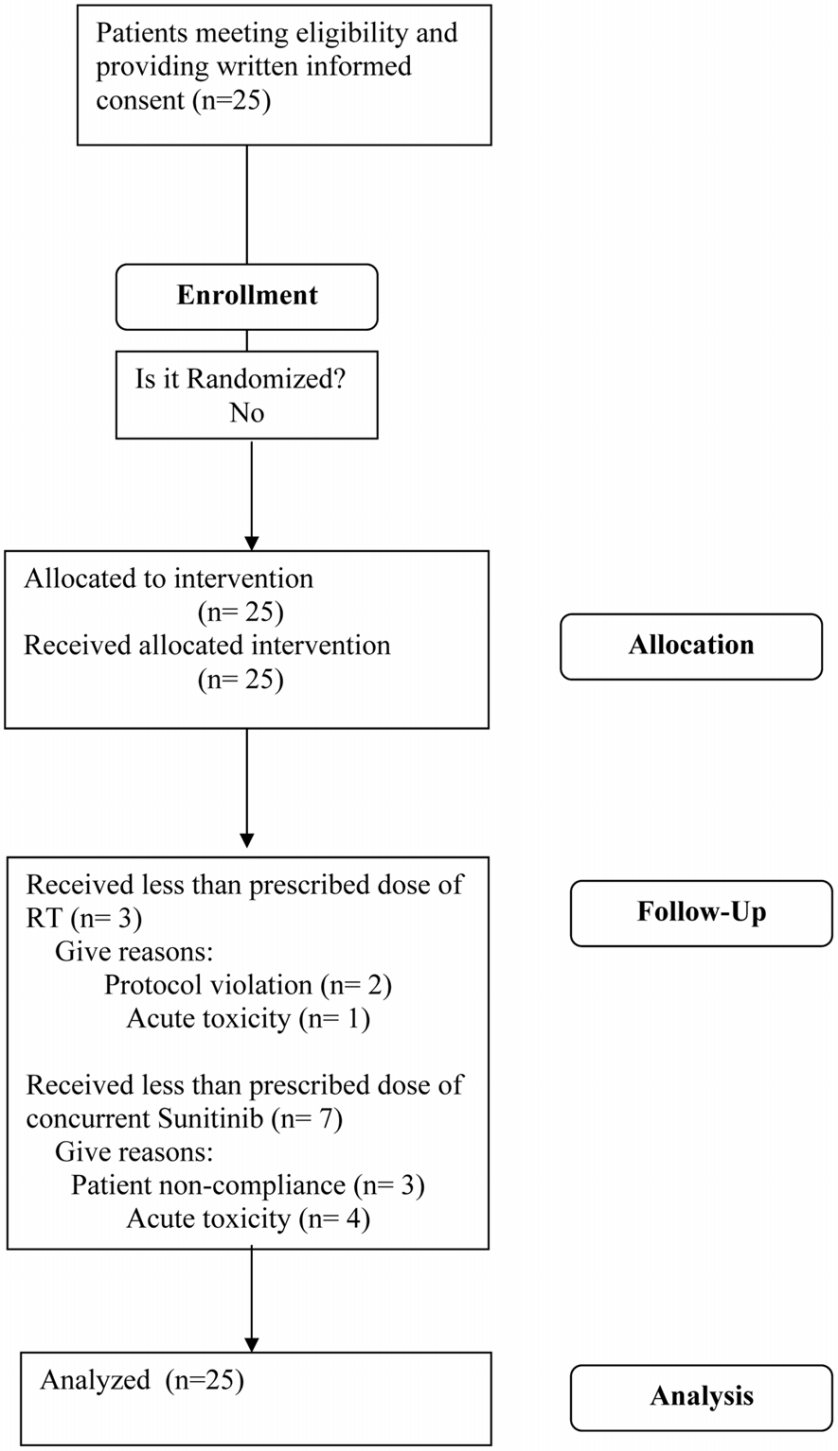
CONSORT flowchart.

**Table 1:** Baseline Patient Characteristics.

Variable	Number (%)
*Median age*	63 (range 54–83)
50–69	16 (64%)
≥70	9 (36%)
*Sex*	
Male	18 (72%)
Female	7 (28%)
*ECOG performance status*	
0	4 (16%)
1	13 (52%)
2	8 (32%)
*Previous chemotherapy*	
No	12 (48%)
Yes	13 (52%)
*Prior RT*	
No	15 (60%)
Yes	10 (40%)
*Number of metastases*	
1	13 (52%)
2	5 (20%)
≥3	7 (28%)
*Largest tumor size*	
≤3 cm	15 (60%)
>3 cm	10 (40%)
*Number of involved organs*	
1	20 (80%)
≥ 2	5 (20%)
*Treatment site*	49 total tumors
Bone	21 (43%)
Lung	14 (29%)
Lymph node	8 (16%)
Visceral (adrenal, thyroid, inferior vena cava, chest wall)	6 (12%)
*Tumor type*	
Head and neck squamous cell carcinoma	4 (16%)
Hepatocellular carcinoma	4 (16%)
Non-small cell lung carcinoma	4 (16%)
Renal cell carcinoma	4 (16%)
Prostate adenocarcinoma	2 (8%)
Colorectal adenocarcinoma	2 (8%)
Pancreatic adenocarcinoma	1 (4%)
Melanoma	1 (4%)
Other (sarcoma, breast, skin squamous cell, parotid, thyroid, small cell lung)	3 (12%)

### Patient Eligibility

Patient eligibility was described previously [14]. Briefly, eligible patients had pathologically confirmed solid tumor malignancy with 1 to 5 sites of active metastatic disease on whole body imaging (PET or CT chest, abdomen, pelvis and bone scan) measuring ≤6 cm. Other key eligibility criteria included age ≥18 years, Eastern Cooperative Oncology Group (ECOG) performance status of 0–2, and adequate hematologic, hepatic and renal function. Eligibility required prior chemotherapy or radiation to be discontinued for at least 2 weeks before study entry. Patients were excluded if they had uncontrolled brain metastases, malignant pleural or pericardial effusion, life expectancy <3 months, prior radiation to targeted area(s) or uncontrolled intercurrent illness. Due to fatal bleeding occurring in a patient receiving anticoagulant therapy, the trial was amended to exclude patients with a history of non-inducible bleeding or who required continuation of anticoagulation during study treatment. Between February 2008 and September 2010, 26 patients were enrolled on the study. One patient withdrew prior to starting treatment due to declining performance status, and was excluded from analysis.

### Drug Administration

Based on phase I data, the phase II regimen of sunitinib was 37.5 mg daily on days 1–28. Sunitinib was administered orally once daily in 6-week cycles consisting of 4 weeks of treatment followed by 2 weeks without treatment. Sunitinib was provided by Pfizer. After completion of concurrent sunitinib and radiotherapy, the treating medical oncologist had the option of continuing on maintenance sunitinib for additional cycles if there was no unacceptable toxicity or progression. If patients did not receive maintenance sunitinib, patients generally received alternative chemotherapy, biological therapy or hormonal therapy, unless limited by age or performance status.

**Figure 2:**
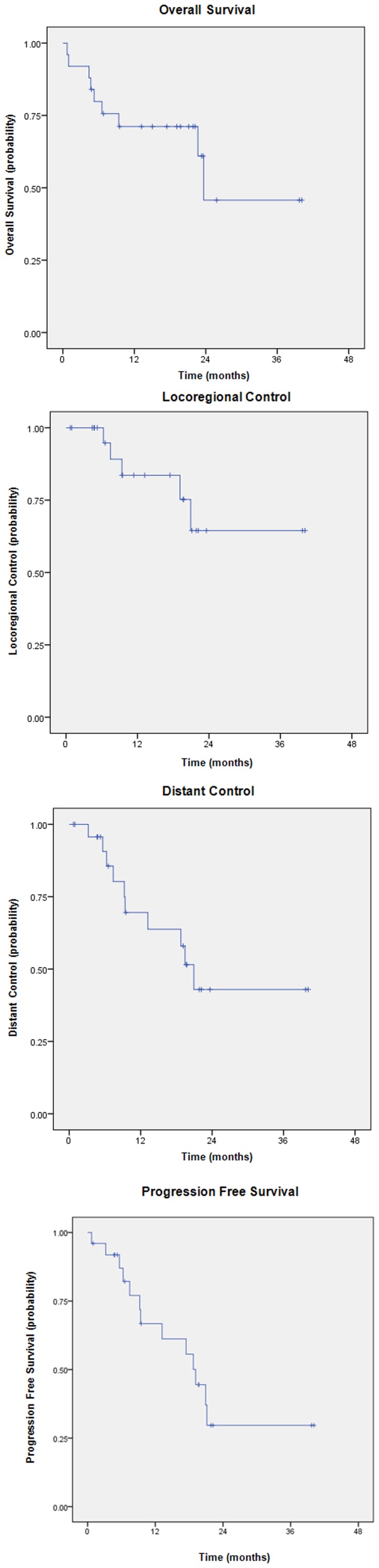
Kaplan-Meier survival curves. a) Overall survival. b) Local control. c) Distant control. d) Progression-free survival.

**Figure 3:**
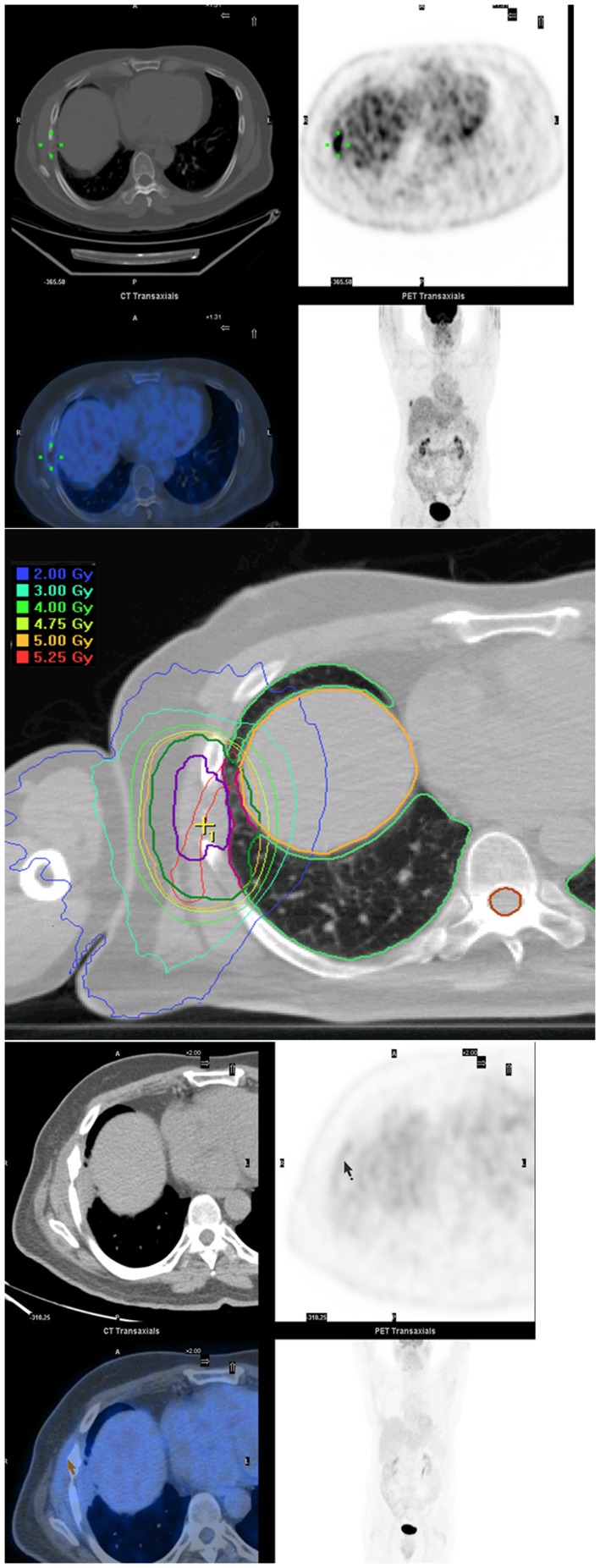
Representative patient treated with concurrent sunitinib and IGRT. a) Pretreatment PET/CT demonstrates a biopsy proven solitary metastasis in the right 7^th^ rib in a patient with non-small cell lung cancer. b) The rib lesion was treated with Novalis using dynamic arcs using the ITV method with an abdominal belt used to dampen respiratory motion. Daily kV imaging was accomplished using bone fusion. There was excellent coverage of the PTV with selective sparing of the normal lung, liver and skin. c) Complete response on PET/CT 23 months after Sutent +RT.

### Radiation Guidelines

Radiation was administered concurrently with the first cycle of sunitinib from days 8–12 and 15–19. Each patient’s treatment was individualized with respect to immobilization and radiation planning technique to optimally cover the target volume and adequately account for organ motion while adhering to strict normal tissue dose and volume limits, as described previously [14]. All patients underwent CT simulation with custom immobilization using an Alpha Cradle, Vac Lock bag or Aquaplast mask. For lung and abdominal tumors, maximum inspiratory, expiratory and free-breathing CT scans were fused to document the maximum amplitude of tumor motion for estimation of an ITV. Relaxed end expiratory breath holding, forced shallow breathing and/or external optical tracking often supplemented with an abdominal belt was utilized for tumors with documented respiratory motion. The gross tumor volume (GTV) was defined as gross tumor on CT, MRI and/or PET. GTV to planning target volume (PTV) expansion ranged from 0.5–1.5 cm, depending on extent of organ motion with consideration for the proximity to critical structures. The recommended phase II dose is 50 Gy in ten fractions over two weeks. Dose was prescribed to the PTV with >90% of the target receiving the prescription dose and a 3D maximum of <110%. When necessary due to the immediate proximity to critical serial structures (e.g., spinal cord, small bowel, esophagus), normal tissue protection was prioritized above target coverage. Planning constraints on organs at risk were described earlier [14]. Treatment planning consisted of conformal arcs, intensity modulated radiation or 3-dimensional forward planning. Daily image guidance was mandatory using implanted fiducial markers or bone fusion.

**Table 2:** Adverse Events.

Adverse Event	All grades	Grade 3	Grade 4	Grade 5
Anemia	18	2	0	0
Neutropenia	14	2	0	0
Fatigue	18	0	0	0
LFT abnormalities	15	1	0	0
Thrombocytopenia	15	4	0	0
Mucositis/stomatitis	8	0	0	0
Nausea/vomiting	7	0	0	0
Skin changes	4	0	0	0
Diarrhea	5	0	0	0
Hypertension	3	0	0	0
Bleeding	4	1	0	1*
Metabolic abnormalities	2	1 (PO_4_)	0	0
Increased creatinine	5	0	0	0

* One case occurred after sunitinib treatment and was likely related to reirradiation performed prior to protocol therapy.

### Follow-up and Study End Points

The primary end point for the phase II trial was PFS measured at 2 years post-therapy. Follow-up visits were planned 1 month after completing radiation therapy (RT) and every 3 months subsequently for 2 years. Patients underwent diagnostic imaging studies before all follow-up visits after the initial 1-month visit. Toxicity was assessed in patients at regular intervals by using the Common Terminology Criteria for Adverse Events (version 3.0). Tumor response was assessed using Response Evaluation and Criteria in Solid Tumors (RECIST), which was modified to incorporate PET/CT information [15]. Local in-field recurrence was defined as progression or recurrence within the high-dose region (>80% isodose volume). Actuarial survival and disease control rates were evaluated by the Kaplan-Meier method. Cause of death was ascertained and attributed to local progression, distant progression, comorbid illness or treatment-related toxicity.

**Table 3:** Trials for Oligometastatic Disease.

Year	Author	Protocol	Patients	Site	No. oflesions	LC	DC	PFS	OS	Reference
2007	Milano	50 Gy in 10 fx	121	Lung, Liver, Brain, Adrenal glands, Bone, Thoracic, pelvic, or abdominal lymph nodes	≤5	2-year 67%;4-year 60%	2-year 34%;4-year 34%	2-year 26%;4-year 20%	2-year 50%;4-year 28%	4
2008	Salama	24–60 Gy in 3–8 fx	29	Lung, Liver, Lymph nodes, Bone, Adrenal, Soft tissue	≤5	Median follow-up of 14.4 mo,79% without progressionin treated site	Median follow-upof 14.4 mo, 45%without distantdiseaseprogression	21% at median of14.4 months	NR	7
2009	Lee	Median dose 41.8 Gyin 6 fx	68	Liver	1–8	1-year 71%	NR	NR	17.6 monthsmediansurvival	3
2009	Rusthoven	Phase I 48–60 Gyin 3 fx; Phase II 60 Gyin 3 fx	38	Lung	1–3	1-year 100%;2-year 96%	NR	Distal progressionin 64% of pts(median4 months)	2-year 55% inpts w/o priorsystemic therapy;2-year 32% inpts with at leastone priorregimen	5
2009	Rusthoven	Phase I 36–60 Gyin 3 fx; Phase II 60 Gyin 3 fx	47	Liver	1–3	1-year 95%;2-year 92%	NR	Distal progressionin 83% of pts(median of 6 months)	2-year 30%	6
2011	Tong	Sunitinib 37.5 mg dailyfor 28 days; 50 Gyin 10 fx	25	Bone, Lung, Lymph nodes, Visceral	≤5	18-month 75%	18-month 52%	18-month56%	18-month 71%	

Abbreviations: LC, local control; DC, distant control; PFS, progression-free survival; OS, overall survival; fx, fractions; NR = not reported.

### Correlative Immune Studies

Peripheral blood mononuclear cells were isolated and analyzed after Ficoll-Hypaque fractionation from 5 patients treated with sunitinib and concurrent IGRT with advanced cancer. Specimens were collected on days 0 (prior to starting sunitinib) and 7 (after 7 days of sunitinib but before starting IGRT). Cells were stained using fluorochrome-conjugated antibodies to identify the following immunophenotypes: CD4+, CD8+ (T cells); HLA-DR+/CD19+/CD86+ (B cells); CD303+/CD123+ (plasmacytoid dendritic cells; pDC); Lin-/HLA-DR-/CD11b+/CD33+ (MDSC); CD14+ (monocytes); CD4+/CD25+/Foxp3+/IL-7 receptor low (Treg). The percent increase or decrease in these cell populations was determined and expression levels of surface markers were quantified by mean fluorescence intensity. The data from individual patients was compared before and after 7 days of sunitinib treatment.

### Statistical Considerations

The primary end point was PFS, measured as time from the initiation of non-surgical treatment until last follow-up or disease progression using intent to treat principles. Failures were scored as local, regional or distant. Overall survival is defined as the proportion of patients who are alive since the start of treatment. Local control is defined as the absence of local failure, with the criteria of (1) an increase in ^18^F-FDG tumor SUV of >25% within tumor region defined on baseline scan, or (2) a visible increase in the extent of ^18^F-FDG tumor uptake (20% in longest dimension). Distant control is defined by the absence of new ^18^F-FDG uptake in metastatic lesions not identified on baseline (pre-treatment) imaging. Progression-free survival is defined as survival in the absence of local or distant progression, with the criteria of (1) an increase in ^18^F-FDG tumor SUV of >25% within tumor region defined on baseline scan, (2) a visible increase in the extent of ^18^F-FDG tumor uptake (20% in longest dimension), or (3) the appearance of new ^18^F-FDG uptake in metastatic lesions not previously identified. For analysis of overall survival and progression-free survival, deaths were considered events.

Statistical analyses and Kaplan-Meier survival curves were calculated using PASW Statistics 18 (IBM SPSS Statistics, Armonk, NY). Tables were generated by Microsoft Excel 2010 (Microsoft, Redmond, WA). Descriptive statistics were used to report the quantitative pre- and post-treatment immune responses (mean±SD, 10^5^ cells per mL). The paired Student’s *t* test was used to compare the groups and *p*≤0.05 was considered to be statistically significant.

## Results

### Patients

Between February 2008 and September 2010, 25 patients with 49 discrete metastases were treated on protocol (Figure 1). The median follow up for surviving patients was 17.5 months (range, 0.7 to 37.4 months). Baseline characteristics for all treated patients are listed in Table 1. The most common tumor types treated were head and neck, liver, lung, kidney, and prostate cancers. The most common sites of metastases treated were bone, lung and distant lymph nodes. Twenty-two patients (88%) received treatment as per protocol. One patient discontinued radiation and sunitinib after a dose of 25 Gy secondary to acute toxicity. Two patients received a reduced dose of 40 Gy due to the judgment of the treating radiation oncologist, and these were classified as protocol violations. Maintenance sunitinib was used in 32% of patients.

### Patterns of Failure and Survival

At last follow-up, 11 (44%) patients were alive without evidence of disease, 7 (28%) were alive with distant metastases, 3 (12%) were dead from distant metastases, 3 (12%) were dead from comorbid illness, and 1 (4%) was dead from treatment-related toxicities. The 18-month estimates for local control and distant control were 75% and 52%, respectively. The 18-month estimates for PFS and OS were 56% and 71%, respectively (Figure 2). The median time to PFS was 9.5 months and the median survival has not been reached. A representative patient treated with concurrent sunitinib and IGRT is shown in Figure 3.

### Toxicity

The most common grade ≥3 acute toxicities were neutropenia, thrombocytopenia, bleeding and liver function test abnormalities. Taken together, 28% of patients experienced at least one grade ≥3 toxicity. Toxicities are described in Table 2. All grade ≥3 events, include one case of grade 5 gastrointestinal hemorrhage, were considered likely related to sunitinib rather than radiotherapy. The 4 deaths attributed to comorbid illness all occurred in patients who discontinued sunitinib for at least 30 days prior to death and were considered unlikely to be related to protocol therapy. These deaths included 2 patients with cardiopulmonary arrest and 1 elderly patient who died peacefully at home. One patient with small cell lung cancer underwent autopsy that demonstrated bronchobiliary fistula outside of the radiation field in a patient who underwent 6 prior lung and liver surgeries. Notably, there was no pathological evidence of residual small cell lung cancer.

### Immune Responses

Compared to pretreatment levels, cancer patients have significantly increased average number of CD4+ T cells after 7 days of sunitinib treatment (3.17±0.92×10^5^ to 3.63±0.84×10^5^; paired t-test, *p* = 0.04). There was a significant decrease in the average of number of Lin-CD33+ MDSC cells (2.20±0.95×10^5^ to 1.51±0.74×10^5^; *p* = 0.02), plasmacytoid dendritic cells (0.03±0.01×10^5^ to 0.02±0.01×10^5^; *p* = 0.01) and T-regulatory cells (0.28±0.05×10^5^ to 0.26±0.04×10^5^; *p* = 0.06). Although an increase in CD8+ T cells was detected in some patients, this failed to reach statistical significance.

## Discussion

In this manuscript, we report results of a prospective phase II trial investigating the efficacy of concurrent sunitinib and hypofractionated IGRT for the treatment of patients with one to five distant metastases from solid tumors. At a median follow-up of 17.5 months, the 18 month PFS was 56%, with 6 patients who remain alive and free of disease progression at 18 to 37+ month follow-up. Various pathologies were represented among long-term survivors, including renal cell carcinoma, hepatocellular carcinoma, hormone-refractory prostate cancer and non-small cell lung cancer. In addition to previously published phase I data, these data support the notion that durable complete clinical and radiographic remissions can be achieved in a subset of patients with oligometastases treated with both local and systemic therapy.

In contrast to published studies investigating radiation alone for oligometastases, concurrent sunitinib and radiation is associated with a higher rate of acute grade ≥3 toxicity [3]–[7]. Although toxicity from sunitinib is generally manageable, serious toxicities, including grade 5 hemorrhage, were noted. Radiation of large volumes of bone marrow and liver can exacerbate hematological toxicities associated with sunitinib. Therefore, although 50 mg is tolerable when sunitinib is administered as monotherapy, when concurrent sunitinib is given with radiation, a reduced daily dose of 37.5 mg is recommended [14]. Further, sunitinib should be used with extreme caution in patients with a history of non-inducible bleeding and patients requiring anticoagulation or antiplatelet medication during treatment [16]. Taken together, concurrent sunitinib and radiation can only be justified if PFS and OS are superior to either sunitinib or radiation alone.

Two recently published clinical trials reported promising rates of PFS in patients with oligometastases treated with radiation alone (see Table 3). The University of Rochester published the largest phase II experience of hypofractionated IGRT for oligometastases mainly treated with 50 Gy in 10 fractions [4]. Milano et al. reported a 2-year PFS of 26%; patients with breast cancers (32% of total patients) had the highest PFS while patients with pancreatic and hepatobiliary tumors had the lowest PFS [4]. A phase I trial of stereotactic body radiotherapy (SBRT) to a dose of 24–60 Gy in 3 fractions at the University of Chicago demonstrated a crude 21% rate of freedom from progression at a median follow-up of 15 months [7]. Although not strictly limited to oligometastases, three recently reported phase I/II trials investigating SBRT (36–60 Gy in 3–6 fractions) for lung and liver metastases demonstrated promising local control rates of 71–96%, although 64–83% developed distant progression at 4–6 month median follow-up [3], [5], [6]. Due to small sample sizes and heterogeneous populations enrolled in these studies, a potential clinical benefit of adding concurrent systemic therapy to radiotherapy for patients with oligometastases cannot be excluded.

Although not directly comparable to studies investigating systemic therapy alone for stage IV cancer, a complete understanding of natural history of metastases treated without local therapy may inform future research. Median PFS with palliative systemic therapy alone for stage IV breast, colorectal, non-small cell lung and hormone-refractory prostate cancers ranges from 2–12 months [17]–[26]. In the two trials with the highest PFS that investigated combinations of biological agents in combination with conventional chemotherapy as first line treatment for breast and colorectal cancer, the 2 year PFS was in the 10–15% range [19], [21]. Median PFS for metastatic renal cell carcinoma with sunitinib was 11 months with a 2-year PFS of less than 20% [27]. A recent analysis of patients with metastatic lung cancer treated with first-line chemotherapy alone demonstrates that the vast majority of patients treated with drug therapy alone ultimately progress, often at sites of initial bulk [28]. These data suggest that sustained long-term remissions are only possible if both local and systemic disease are adequately treated. However, a randomized trial is necessary to definitively demonstrate a benefit for concurrent sunitinib and radiation, compared to either treatment alone.

Although combining radiation with angiogenesis inhibitors has been extensively studied in animal models, a significant benefit for local control or survival has not yet been shown in humans [29]. This study demonstrates the feasibility of combining sunitinib, a multitargeted tyrosine kinase angiogenesis inhibitor, with radiotherapy to treat tumors occurring at various sites throughout the body. The finding that some patients remain free of distant progression raises the possibility that even a brief course of sunitinib during radiotherapy may offer some protective effect on micrometastases. Extensive preclinical data and rapidly accumulating human data suggests that sunitinib decreases immunosuppressive MDSC and Treg cells [13], [30]. While total body radiation is immunosuppressive, accumulating preclinical data suggests that local radiation may enhance antitumor immunity by priming the tumor microenvironment [31]. Most recently reported by Lee et al., mice that received ablative radiotherapy were found to have dramatically increased T cells in draining lymphoid tissues, leading to reduction in primary tumor burden or distant metastases in a CD8+ T cell-dependent fashion [32]. Thus, concurrent sunitinib and local radiotherapy may serve as a platform to improve existing immunotherapeutic approaches. Further analysis of patients treated with concurrent sunitinib and radiotherapy is underway in our laboratory. Additionally, bone marrow derived cells, including MDSC, have been implicated in tumor vasculogenesis and resistance to angiogenesis inhibitors and radiotherapy [33].

In summary, concurrent sunitinib and image-guided radiotherapy represents a novel approach to the treatment of patients with oligometastases that warrants further clinical and translational study.

## Supporting Information

Checklist S1 CONSORT Checklist
(DOC)

Protocol S1 Trial Protocol
(DOC)
