# Supplementary material for: Phase II Trial of Concurrent Sunitinib and Image-Guided Radiotherapy for Oligometastases
Source: PLoS One. 2012 Jun 27;7(6):e36979. doi: 10.1371/journal.pone.0036979 (PMC3384658; doi:10.1371/journal.pone.0036979)
Supplement: Protocol S1 — Trial Protocol (DOC) [file pone.0036979.s002.doc]

**Phase I/II Study of Stereotactic Radiation Therapy and Concurrent and Adjuvant Sutent (SU11248) as Treatment for Oligometastatic Disease**

Study Number: MSH 06-0906

Principal Investigator, Radiation Oncology Chair: Johnny Kao, M.D.

Medical Oncology Chair: Stuart Packer, M.D.

Co-Investigators:

Max Sung, M.D.

Myron Schwartz, M.D.

Richard Stock, M.D.

Huma Syed, M.D.

Vishal Gupta, M.D.

Support Provided in Part by Pfizer.

Version 12/16/09

**Phase I/II Study of Stereotactic Radiation Therapy and Concurrent and Adjuvant Sutent (SU11248) as Treatment for Oligometastatic Disease**

**Principal Investigator**: Johnny Kao, M.D.

Mount Sinai School of Medicine

Department of Radiation Oncology

1 Gustave Levy Place, Box 1236

New York, NY 10029

johnny.kao@mountsinai.org

(212) 241-7503

**Trial Center and number of patients planned:** 52 patients

**Trial period**:

Estimated date first patient enrolled: 1/1/2007

Estimated date last patient enrolled: 9/1/2011

**Endpoints:**

- Phase I: The primary objective is to establish the maximum tolerated dose (MTD) of the combination of extracranial stereotactic radiation (Gy) and Sutent (mg), the dose limiting toxicity (DLT) of the proposed regimen, and to determine a recommended phase II dose (RPTD) of extracranial stereotactic radiation therapy in combination with Sutent.
- Phase II: The primary objectives are to determine the progression free survival and overall survival of patients treated with the RPTD.

**Trial Design:**

A phase I trial will be performed using escalating doses of extracranial stereotactic radiation therapy and Sutent to all involved sites of disease in patients with metastatic cancer with ≤ five disease sites. If a RPTD regimen is identified, additional patients will be treated on a phase II trial to determine the efficacy of this protocol.

**Background and Rationale**:

Patients with metastatic cancer are generally treated with chemotherapy, which has improved median survival compared to best supportive care. Despite this, patients continue to have persistent disease at sites that were initially involved with cancer.

Radiation therapy is an effective modality for treating localized cancer but until recently has been only used for palliation of symptoms once a patient develops metastatic disease. However, there is a rapidly enlarging clinical experience of extracranial stereotactic radiation to aggressively treat metastatic sites. Since patients with metastatic disease clearly have systemic disease, there is an urgent need to integrate effective drug therapy with increasingly effective local therapy. Although, both highly focused radiation and a targeted biological therapy such as Sutent have favorable toxicity profiles, there are no clinical data on the combination. The goal of this trial is to use increasing doses of extracranial stereotactic radiation therapy to all sites of involved disease in combination with increasing doses of Sutent in order to determine the safety and efficacy of this regimen. Ultimately, the goal would be to determine if the addition of radiation therapy and Sutent will prevent further progression of disease in the involved sites and possibly improve overall survival.

**Patient population:**

- Patients with metastatic cancer without pleural/pericardial effusion
- Patients with 1-5 sites of involved disease with each individual site </= 6 cm maximum diameter or 500 cc volume (gross tumor volume) as seen on standard imaging (CT, MRI, bone scan).

**Inclusion criteria:**

- Histologically or cytologically confirmed diagnosis of cancer with metastatic disease
- No prior RT to currently involved sites
- Informed consent
- Room air saturation (SaO2) > 90%
- ECOG performance status < 2

**Treatment Plan**:

- Sutent be administered PO QD from days 1 to 28 (see Figure 1)

Figure 1. Treatment Schema

Determine eligibility

**↓**

| **REGISTER** | **Treatment** | Days  1-7 | 8-12 | 13-14 | 15-19 | 20-28 | 29-42 |
| --- | --- | --- | --- | --- | --- | --- | --- |
|  | XRT |  | X |  | X |  |  |
|  | Sutent | X | X | X | X | X |  |

**↓**

Additional chemotherapy (as determined by Medical Oncology)

**↓**

Maintenance Sutent (until death or progression) and appropriate follow-up

- Radiation therapy will start on day 8 and will be given to each site for ten fractions
- Fractions must be separated by at least 16 hours
- The MTD of radiation in combination with Sutent will be determined
- Doses of radiation and Sutent will increase as shown in Table 1 in order to determine the MTD of radiation in Gy and Sutent in mg
- The RPTD will be one dose level lower than the MTD. If the MTD is not reached at dose level 5, no further escalation will occur and this will be the RPTD. The RPTD will then be used for future phase II trials.
- Starting day 43, chemotherapy may be given, as determined by the treating medical oncologist.
- Two weeks after completion of any chemotherapy, maintenance Sutent in 6 week cycles (consisting of Sutent 50 mg PO QD weeks 1-4 followed by no treatment weeks 5-6) until progression or death
- If no chemotherapy is planned, maintenance Sutent (as described above) will start on day 43.

**Table 1. Phase I Dose Levels**

| **Dose Level** | **Radiation Dose** | **Sutent Dose** |
| --- | --- | --- |
| 1 | 40 Gy in 10 fractions | 25 mg PO QD |
| 2 | 40 Gy in 10 fractions | 37.5 mg PO QD |
| 3 | 50 Gy in 10 fractions | 37.5 mg PO QD |
| 4 | 50 Gy in 10 fractions | 50 mg PO QD |
| 5 | 60 Gy in 10 fractions | 50 mg PO QD |

**Duration of protocol treatment:**

Until progression or death

**Number of patients expected to be enrolled each month:**

1-3

**Statistical analysis:**

*Phase I: Feasibility and toxicity.* Patients will receive concurrent treatment with extracranial radiosurgery and Sutent. Both the radiation dose and Sutent dose will be escalated as the trial progresses from Levels 1 through 5 as shown in Table 1. The starting radiation dose is 40 Gy in 10 fractions and the starting Sutent dose is 25 mg PO QD. The dose escalation strategy is as follows: The trial will start with dose level 1. At each level, 3 patients will be treated and their toxicities will be assessed. If no patients experience dose limiting toxicity, the trial will proceed to the next dose level. If 1 patient develops unexpected acute toxicity at any dose level, an additional 3 patients will be treated at that dose level. If ≥2 patients experience unexpected toxicity, the previous dose level will be declared the maximal tolerated dose for Phase II evaluation

*Phase II (Includes 3 to 6 patients from Phase I MTD):* The median progression free survival with optimal chemotherapy alone for the 4 most common tumor types (lung, breast, prostate and colorectal) ranges from 2.2 to 10.6 months, depending on primary tumor site and degree of pretreatment. Therefore, we will assume a median time to progression of 6.4 months (standard deviation 3.2 months) with chemotherapy alone. Using the data from this trial, response rates will be estimated and Kaplan-Meier curves generated for progression-free and overall survival (1). With 38 patients for the phase II portion, this study has 87% power (α=0.05) to detect a 25% increase in time to progression. Also the 2 year freedom from progression is <5% with chemotherapy therapy and this study has 70% power (α=0.05) to demonstrate a 10% absolute increase in 2 year progression-free survival.

**Table 1: Pre-therapy and weekly checklists during RT and for follow-up**

| Item | Pre-therapy | Week 1 RT | Week 2 RT | Week 3 RT1 | Week 4 RT1 | Follow-up7 |
| --- | --- | --- | --- | --- | --- | --- |
| Inclusion/exclusion criteria | X |  |  |  |  |  |
| History and Physical | X | X | X | X | X | X |
| Informed consent | X |  |  |  |  |  |
| Pathology confirming cancer | X |  |  |  |  |  |
| Documentation of metastatic disease | X |  |  |  |  |  |
| Performance status | X |  |  |  |  |  |
| Adverse effect assessment |  | X | X | X | X | X |
| CT scan of chest/abdomen/pelvis or whole body PET/CT2 | X |  |  |  |  | X8 |
| Bone scan (omit if PET scan performed)2 | X |  |  |  |  | X8,9 |
| Plain X-ray and/or CT and/or MRI of bone abnormality seen on bone scan | X |  |  |  |  | X3 |
| MRI brain | X3 |  |  |  |  | X3 |
| Hematology4 | X | X | X | X | X | X7 |
| Biochemistry5 | X |  |  |  |  | X7 |
| Pregnancy test6 | X |  |  |  |  |  |

1  If applicable

2  The same test should be done throughout therapy and follow-up.

3 If clinically indicated

4  CBC, differential, and platelet count

5  Comprehensive metabolic profile (glucose, sodium, potassium, chloride, carbon dioxide, BUN, creatinine, calcium, total bilirubin, alkaline phosphatase, AST, ALT)

6  For women of child bearing potential, serum hCG

7  Follow-up visits including hematology and biochemistry tests: every 2 weeks for 2 months then every 3 months for 1 year then every 6 months indefinitely

8  Initially 4-6 weeks after completion of radiation therapy to asses response; then every 3 months for first year then every 6 months indefinitely

­9Optional if not previously involved

Abbreviations: CBC= complete blood count, CT= computed tomography, MRI= magnetic resonance imaging

| **TABLE OF CONTENTS** |  |
| --- | --- |

Page

Eligibility Summary 9

Eligibility Checklist 10

1. Objectives 12
2. Introduction/Background 12
3. Trial Schema 15
4. Inclusion Criteria 16
5. Exclusion Criteria 17
6. Criteria for Discontinuation/Withdrawal of Informed Consent 18
7. Treatment Plan – Pre-radiation, Peri-radiation and Post-radiation Assessments 18
8. Treatment Plan – Extracranial Stereotactic Radiation Therapy 21
9. Drug Therapy - Sutent 23
10. Phase I Dose Escalation Rules 26
11. Additional Interventions – Guidelines for Chemotherapy, Surgery and/or Additional Radiation 27
12. Toxicity 27
13. Measurement of Effect 28
14. Biological Correlates 30
15. Adverse Events 31
16. Statistical Considerations 33
17. Confidentiality 34
18. References 34
19. Appendix I: Informed Consent Form 38
20. Appendix II: Performance Status Scales 39
21. Appendix III: Mount Sinai Department of Radiation Oncology Clinical Trials Data and Safety Monitoring Plan 40
22. Appendix IV: Standard Operating Procedures for Blood and Urine Collection and Storage 52

**ELIGIBILITY SUMMARY:**

Inclusion Criteria *(e.g., performance status, organ function)*:

- Zubrod Performance Scale 0-1
- Metastatic disease confirmed by biopsy or imaging
- 5 or fewer sites of metastatic disease on tumor staging (either CT chest/abdomen/pelvis plus bone scan or whole body FDG-PET)
- All tumors measure ≤ 6 cm
- Age ≥ 18
- Chemotherapy must be completed at least 2 weeks prior to radiation
- Signed informed consent
- Adequate bone marrow function, defined as follows:

- Platelets > 100,000 cells/mm3based upon CBC/differential obtained within 2 weeks prior to registration on study

- Absolute neutrophil count (ANC) > 1,800 cells/mm3 based on CBC/differential obtained within 2 weeks prior to registration on study

- Hemoglobin > 8.0 g/dl based upon CBC/differential obtained within 2 weeks prior to registration on study (Note: The use of transfusion or other intervention to achieve Hgb > 8.0 g/dl is acceptable.)

**ELIGIBILITY CHECK LIST**

The following questions will be asked at Study Registration:

______ Name of institutional person registering this case?

______ Has the eligibility checklist been completed?

______ Is the patient eligible for this study?

______ Date the study-specific Consent Form was signed? (must be prior to study entry)

______ Patient’s Initials (First Middle Last) [If no middle initial, use hyphen]

______ Verifying Physician

______ Patient’s ID Number

______ Date of Birth

______ Race

______ Ethnic Category (Hispanic or Latino; Not Hispanic or Latino; Unknown)

______ Gender

______ Patient’s Country of Residence

______ Zip Code (U.S. Residents)

______ Patient’s Insurance Status

______ Will any component of the patient’s care be given at a military or VA facility?

______ Treatment Start Date

______ Blood kept for cancer research?

______ Blood kept for medical research?

______ Allow contact for future research?

______ Is there a biopsy proven malignancy?

______ Was a PET/CT or CT/MRI chest/abdomen/pelvis AND bone scan performed?

______ Specify the date of CT, MRI, bone scan or PET/CT (mm-dd-yyyy)

______ Number of measurable lesions on the CT, MRI, bone scan or PET/CT?

______ Size of largest lesion?

______ Are all visible lesions treated within the radiation fields according to protocol criteria.?

______ If not, are these lesions being treated with an alternate modality (surgery, radiofrequency ablation, photodynamic therapy, focused ultrasound, intracranial radiosurgery, standard fractionation radiation therapy)

______ Age?

______ Zubrod Performance Status?

______ Results of ANC (cells/mm3)

______ Date of ANC (mm-dd-yyyy)

______ Results of platelet count (cells/mmз)

______ Date of platelet count (mm-dd-yyyy)

______ Results of hemoglobin (g/dl)

______ Date of Hemoglobin (mm-dd-yyyy)

______ Was an INR, total bilirubin, albumin, alkaline phosphatase, ALT and AST drawn within 2 weeks prior to study entry?

______ Date additional pretreatment labs were drawn? (mm-dd-yyyy)

______ Does the patient have any history of non-inducible bleeding? (12/16/09)

______ If the patient is on any blood thinner (Coumadin, lovenox, aspirin, plavix, NSAIDs, etc) can this be discontinued prior to starting protocol therapy? (12/16/09)

______ Was Chest CT or PET/CT performed within 30 days of study entry?

______ Was a negative serum pregnancy test obtained within 72 hours prior to registration?

______ Are any tumor markers drawn and found to be positive (ie., CEA, AFP,

______ Has the patient received prior chemotherapy?

______ IF YES Completion date of chemotherapy (mm-dd-yyyy)

______ Has the patient had a prior invasive malignancy?

______ IF YES Date of eradication of prior malignancy (mm-dd-yyyy)

______ Has the patient received prior RT to the region of the study cancer that would result in overlap of radiation fields?

______ Has the patient had unstable angina and/or CHF requiring hospitalization within the last 6 months?

______ Has the patient had a transmural MI within the last 6 months?

______ Has the patient had an acute bacterial or fungal infection requiring antibiotics at the time of registration?

______ Has the patient had a COPD exacerbation or other respiratory illness requiring hospitalization or precluding study therapy at the time of registration?

______ Does the patient have active hepatitis or clinically significant liver failure?

______ Is there CNS metastasis?

______ Is the patient, male or female, of reproductive potential?

______ Will a medically acceptable form of contraception be used?

Eligibility checklist completed by_____________________

Date Completed:______________________

**1. OBJECTIVES**

- Phase I: The primary objective is to establish the maximum tolerated dose (MTD) of the combination of extracranial stereotactic radiation (Gy) and Sutent (mg), the dose limiting toxicity (DLT) of the proposed regimen, and to determine a recommended phase II dose (RPTD) of extracranial stereotactic radiation therapy in combination with Sutent.
- Phase II: The primary objectives are to determine the progression free survival and overall survival of patients treated with the RPTD. Secondary endpoints are response rates, quality of life and symptom relief of patients treated with the RPTD.

**2. INTRODUCTION/BACKGROUND**

2a. Rationale for New Approaches to Metastatic Cancer

Cancer is the second leading cause of death in the United States, predominantly due to inability to control progressive metastatic disease (2). Save for notable exceptions such as germ cell tumors, drug therapy alone is not curative therapy for patients with solid tumors with gross disease (3). However, not all patients with metastatic cancer succumb to their disease. Surgery and chemotherapy has resulted in long-term disease free survival in approximately 25% of patients with colorectal cancer and isolated liver metastases (4). Additionally, some patients with isolated pulmonary metastases, most notably soft tissue sarcoma, are rendered disease-free with resection and chemotherapy (5). These data lend support to the notion that not all metastases are diffuse and some patients may limited, organ specific metastases (6).

Spread of the primary tumor to regional lymph nodes or distant organs is common in patients with cancer. The evolution of clinical metastasis is based in part on the anatomic proximity of the primary tumor to the lymphatic and vascular supply and drainage. Examples include the spread of epidermoid cancers of the lung, head and neck and cervix to draining regional lymph nodes and gastrointestinal cancers to the liver through the portal system. Historically several hypotheses based on clinical evidence attempted to explain mechanisms of metastatic tumor cell spread. Halsted suggested that tumor cells spread in an orderly manner initially spreading to regional lymph nodes and then centrifugally to distant organs (7). In contrast, the “systemic hypothesis” first suggested by Keynes and most clearly articulated by Fisher proposes two principal types of cancer: those that cannot metastasize and those that have metastasized widely before clinical detection (8, 9). In an attempt to reconcile clinical and laboratory features in a unified hypothesis, the “spectrum” model has been proposed by Hellman (10). This theory accepts that some tumors have spread widely before clinical detectability while others never metastasize, but for the majority of cancers, metastatic capacity evolves during the clinical phase of tumor growth. In this model, metastatic proclivity evolves during tumor growth. During this evolutionary process, there may be a stage, termed “oligometastases,” when metastases are limited in number and location because metastatic capacity has not fully evolved (6). If this hypothesis is valid, these patients would benefit from effective local therapy in addition to systemic therapy.

Recent advances in cytotoxic chemotherapy and biological therapy have resulted in improved overall survival and disease free survival for the most common metastatic cancers including breast, prostate, colorectal and lung cancer (11-15). However, there is no evidence that chemotherapy or biological therapy alone results in an appreciable long-term cure rate. Additionally, the median progression-free survival for the four most common tumor types with chemotherapy alone for the 4 most common tumor types (lung, breast, prostate and colorectal) ranges from 2.2 to 10.6 months, depending on primary tumor site and degree of pretreatment. While conventional chemotherapy has been limited by low therapeutic index, poor drug penetration through tissue and multidrug resistance, targeted therapies are hindered by genetic heterogeneity within tumors and the multiple genetic abnormalities associated with solid tumors (16-18). Therefore, combining targeted and broad spectrum anti-neoplastic strategies are likely needed to significantly improve outcomes in patients with metastatic disease. In a recent patterns of failure analysis, 38 patients with metastatic lung cancer treated on a phase II trial of oxaliplatin and paclitaxel were carefully analyzed. Fifty percent of patients presented with ≤ 3 metastatic sites and 50% had stable disease or progressed only within initially involved sites on follow up, suggesting a potential benefit for local therapy in selected patients (19).

If systemic therapy can control microscopic but not gross disease, adding effective local therapy will impact survival (6). This paradigm was clearly demonstrated in post-mastectomy radiation trials, where radiation improved survival only recent randomized studies that employed active chemotherapy regimens (20, 21). With advances in radiation planning and tumor imaging, it is now possible to safely target sites of gross metastatic disease with high dose radiation therapy (22-26). We hypothesize that combining effective local therapy to gross disease with effective biological therapy for micrometastatic disease represents a potentially curative approach. To our knowledge, there are no published reports of this novel approach.

2b. Extracranial Stereotactic Radiation Therapy

Standard radiation for metastatic disease is given primarily with the goal of palliation (22). Therefore, low dose-intensity radiation is used to avoid treatment-related complications with standard low-precision large field radiation. However, when radiation is used to eradicate gross disease, higher doses are needed. There is now a significant experience in extracranial stereotactic radiotherapy as effective local therapy for metastatic lesions. This technique requires secure patient immobilization, accurate patient repositioning, accounting for internal organ motion, use of highly conformal dose distributions, registration of patient anatomy to fiducial markers and use of dose intense fractionation schemes (24). Local control in excess of 80% has been reported for metastatic tumors of the spine, lung and liver, which is significantly higher than standard radiation (24). Toxicity has been minimal in multiple U.S., European and Japanese trials of extracranial stereotactic radiotherapy to the lung, liver, spine, pelvis and abdomen despite the use very high biological equivalent doses for patients with both organ confined and metastatic cancer (23, 24, 26-31). These data strongly support the use of high dose intensity, focused stereotactic radiation as a safe and locally effective treatment.

2c. Sutent (SU11248) for Advanced Cancers

Sutent (SU11248) is a small molecule receptor tyrosine kinase inhibitor that targets multiple pathways including PDGFRα, c-kit, VEGFR1, VEGFR2, VEGFR3, FLT3 and RET. These tyrosine kinases are implicated in tumor proliferation, angiogenesis and metastasis. As of December 2005, 1563 patients have received at least one dose of Sutent. Overall, Sutent has a favorable toxicity profile with adverse events that have been manageable and reversible. Specifics of Sutent related safety data are described in Section 9.

Sutent significantly prolonged time to progression (27 vs. 6 weeks, p<0.001) and improved overall survival (HR 0.49, p=0.007) in a phase III trial of metastatic GIST following failure of imitanib (32). Additionally in two phase II studies, Sutent had an unprecedented 40% objective response rate in cytokine-refractory metastatic renal cell carcinoma (33). Based on these data, Sutent has been approved by the F.D.A. as treatment for GIST whose disease has progressed despite Gleevec or for patients unable to receive Gleevec. Simultaneously, the F.D.A. approved Sutent for treatment of metastatic renal cell carcionoma. In a phase I trial of Sutent in advanced cancers, 50 mg/day was the recommended maximum tolerated dose, with an objective response rate of 21% (34). Sutent is currently undergoing clinical evaluation in multiple tumor types including breast cancer, non-small cell lung cancer, colorectal cancer and prostate cancer. Trials are underway, which will evaluate the efficacy of Sutent in combination with a number of cytotoxic and biological agents including trastuzuzmab, paclitaxel, docetaxel, eroltinib, carboplatin, cisplatin, gemcitabine, irinotecan, oxaliplatin, 5-fluorocuracil, and capecitabine. However, to date, there are no reported clinical studies of Sutent with radiation.

Angiogenesis inhibitors, including SU11248, have been shown to significantly enhance radiation response by selectively targeting tumor vasculature in preclinical studies (35, 36). In a xenograft model, mice harboring Lewis Lung Cancer or GL261 glioblastoma cells were treated with control, 21 Gy in 3 Gy daily fractions, SU11248 40 mg/kg intraperitoneal injection or concurrent radiation and Sutent. There was significant tumor growth delay with combined radiation and Sutent compared to either treatment alone in both xenograft models (p<0.05). Time to doubling of LLC tumor size was 6-8 days with radiation or Sutent alone vs. 16 days with the combination (35). However, tumors treated with Sutent and radiation eventually regrew unless maintenance Sutent (20 mg/kg intraperitoneal injection BID) was added. These preclinical studies suggest that the combination of radiation and angiogenesis inhibitors, including Sutent preferentially targets tumor vasculature (35, 36). Preliminary data evaluating radiation in combination with angiogenesis inhibitors suggests no overlapping toxicity (37). We propose Sutent in combination with radiation therapy with the goal of improving overall survival in selected patients with limited metastatic disease, as a novel treatment paradigm, irrespective of the primary site.

**Study Proposal and Rationale**

Patients with metastatic cancer are generally treated with chemotherapy, which has improved median survival compared to best supportive care. Despite this, patients continue to have persistent disease at sites that were initially involved with cancer.

Radiation therapy is an effective modality for treating localized cancer but until recently has been only used for palliation of symptoms once a patient develops metastatic disease. However, there is a rapidly enlarging clinical experience of extracranial stereotactic radiation to aggressively treat metastatic sites. Since patients with metastatic disease clearly have systemic disease, there is an urgent need to integrate effective drug therapy with increasingly effective local therapy. Although, both extracranial stereotactic radiation and Sutent have favorable toxicity profiles, there are no clinical data on the combination. The goal of the phase I trial is to use increasing doses of extracranial stereotactic radiation therapy to all sites of involved disease in combination with increasing doses of Sutent in order to determine the dose limiting toxicity (DLT), and maximum tolerated dose (MTD). The goal of the phase II trial is to determine if the addition of radiation therapy and Sutent will improve progression-free survival and overall survival and at the minimum have a positive impact on quality of life, response rate and palliation of symptoms.

**3.** **TRIAL SCHEMA**

Determine eligibility

**↓**

| **REGISTER** | **Treatment** | Days  1-7 | 8-12 | 13-14 | 15-19 | 20-28 | 29-42 |
| --- | --- | --- | --- | --- | --- | --- | --- |
|  | XRT |  | X |  | X |  |  |
|  | Sutent | X | X | X | X | X |  |

**↓**

Additional chemotherapy (as determined by Medical Oncology)

**↓**

Maintenance Sutent (until death or progression) and appropriate follow-up

**Phase I Dose Levels**

| **Dose Level** | **Radiation Dose** | **Sutent Dose** |
| --- | --- | --- |
| 1 | 40 Gy in 10 fractions | 25 mg PO QD |
| 2 | 40 Gy in 10 fractions | 37.5 mg PO QD |
| 3 | 50 Gy in 10 fractions | 37.5 mg PO QD |
| 4 | 50 Gy in 10 fractions | 50 mg PO QD |
| 5 | 60 Gy in 10 fractions | 50 mg PO QD |

- Two weeks after completion of any chemotherapy, maintenance Sutent in 6 week cycles (consisting of Sutent 50 mg PO QD weeks 1-4 followed by no treatment weeks 5-6) until progression or death

**4.** **INCLUSION CRITERIA**

1. Histologically or cytologically confirmed diagnosis of cancer (including epithelial carcinoma, sarcoma, and melanoma)
2. Age 18 years or older
3. Life expectancy > 3 months
4. Patients with AJCC (6th edition, 2002) stage IV cancer with distant metastases and without pleural or pericardial effusion at diagnosis and before start of study
   1. Patients with pleural effusion that is transudative, cytologically negative,

and non-bloody are eligible

- 1. If a pleural effusion is too small for diagnostic thoracentesis, the patient will be eligible.

1. Patients with 1-5 sites of maximum tumor dimension (for each individual site) of ≤ 10 cm or < 500 cc volume and amenable to radiation therapy as seen on standard imaging (CT, MRI, bone scan, PET/CT)
2. Unidimensionally measurable disease (based on RECIST) is desirable but not strictly required.
3. Brain metastases must have been treated prior to enrollment on study, preferably with stereotactic radiosurgery or surgery.
4. Eastern Cooperative Oncology Group (ECOG) performance status ≤ 2 (Karnofsky ≥ 60%)
5. No prior radiation therapy to currently involved tumor sites
6. Room air saturation (SaO2) > 90%
7. Patients must have normal organ and marrow function as defined below:

Hemoglobin > 9.0 g/dl

Absolute neutrophil count (ANC) ≥ 1,500/μl

Platelets ≥ 100,000/μl

Total bilirubin within institutional limits

Albumin > 2.9 g/dl

Alkaline phosphatase < 2.5x upper limit of normal

AST and ALT <2.5 x upper limit of normal

1. Informed consent must be obtained from all patients prior to beginning therapy. Patients should have the ability to understand and the willingness to sign a written informed consent document.
2. Inclusion of Women and Minorities: Both men and women of all ethnic groups are eligible for this trial. The proposed study population is in the table below:

Table 2:

|  | White, not of Hispanic origin | Black, not of Hispanic origin | Hispanic | Asian or Pacific Islander | Unknown | Total |
| --- | --- | --- | --- | --- | --- | --- |
| Male | 24 | 4 | 6 | 2 | 0 | 36 |
| Female | 20 | 3 | 5 | 1 | 0 | 29 |
| Total | 44 | 7 | 11 | 3 | 0 | 65 |

1. Radiation therapy requirements: Patient must have a completed treatment plan approved by the protocol review team (Johnny Kao or Richard Stock AND Vishruta Dumane or Yeh-Chi Lo)

**5.** **EXCLUSION CRITERIA**

1. Other coexisting malignancies or malignancies diagnosed within the previous 3 years with the exception of basal cell carcinoma, cervical carcinoma in situ, and other treated malignancies with no evidence of disease for at least 3 years
2. Uncontrolled intercurrent illness including, but not limited to, ongoing active infection, symptomatic congestive heart failure (CHF), unstable angina pectoris, cardiac arrhythmia, or psychiatric illness/social situations that would limit compliance with the study requirements
3. Patients with clinically significant pulmonary dysfunction, cardiomyopathy, or any history of clinically significant CHF are excluded. The exclusion of patients with active coronary heart disease will be at the discretion of the attending physician.
4. Patients with exudative, bloody, or cytologically malignant effusions are not eligible.
5. Pregnancy or breast feeding (Women of child-bearing potential are eligible, but must consent to using effective contraception during therapy and for at least 3 months after completing therapy)
6. Patients must have no uncontrolled active infection other than that not curable without treatment of their cancer.
7. Prior radiation to target area
8. Patient may not be receiving any other investigational agents during radiotherapy.
9. Prior history of non-inducible bleeding (12/16/09).
10. Requirement for continuation of anticoagulation (defined as Coumadin, lovenox, heparin, plavix, aspirin, NSAIDs or similar drugs) during treatment (12/16/09)

**6. CRITERIA FOR DISCONTINUATION/WITHDRAWAL OF INFORMED CONSENT**

Patients may be discontinued from trial treatment and assessments at any time, at the discretion of the investigator(s). Specific reasons for discontinuing a patient from study treatment are

- objective progression of disease
- patient lost to follow-up (i.e., dropouts)
- adverse events
- protocol non-compliance
- withdrawal of consent

If the reason for withdrawal from the trial is the death of the patient, the two options for categorizing withdrawal are either progressive disease or an adverse event (AE; more than one AE may be documented as a reason for withdrawal). Only one event will be captured as the cause of death. Note that death is an outcome and not an AE.

All deaths that occur within the trial period or within 30 days after administration of the last dose of radiation therapy must be reported primarily for the purposes of serious adverse event (SAE) reporting; however, deaths due unequivocally to progression are not SAEs.

All trial treatment-related toxicities and SAEs must be followed up until resolution.

All patients who have new or worsening grade 3 or 4 laboratory values (Common Terminology Criteria for Adverse Events (CTCAE)) at the time of withdrawal must have additional testing performed, and the results must be recorded in the patients’ medical records. These patients should be followed until the laboratory values have returned to CTCAE grade 1 or 2, or until 30 days after the date of withdrawal (whichever comes first), unless these values are not likely to improve because of the underlying disease. In these cases, the investigators must record their opinions in the patients’ medical records. Laboratory abnormalities should not be reported as adverse events unless any criterion for an SAE is fulfilled, the laboratory abnormality causes the patient to discontinue from the study, or the investigator insists the abnormality should be reported as an AE.

After withdrawal from treatment, unless the patient withdraws consent, follow-up should continue for determination of adverse events, progression-free survival, and overall survival.

**7.** **TREATMENT PLAN: PRE-THERAPY, PERI-THERAPY AND POST-THERAPY ASSESSMENTS**

7.1 Pre-therapy checklist:

- Inclusion/exclusion criteria
- History and physical
- Informed consent
- Pathology confirming cancer
- Documentation of metastatic disease
- Performance status
- Documentation of current medications
- Adverse effect assessment
- CT scan of chest, abdomen, and pelvis
- Bone scan
- Plain X-ray and/or CT and/or MRI of bone abnormality seen on bone scan
- Whole body PET/CT is acceptable replacement for CT scan of chest, abdomen and pelvis and bone scan but is not required
- MRI brain if clinically indicated
- Hematology: CBC (hemoglobin, white blood cell count, platelets) with differential
- Biochemistry: Comprehensive metabolic profile (glucose, sodium, potassium, chloride, carbon dioxide, BUN, creatinine, calcium, total bilirubin, alkaline phosphatase, AST, ALT)
- If suspected to be elevated, obtaining appropriate tumor markers are recommended (PSA, CEA, CA 19-9, EBV viral load, AFP, Beta-HCG)
- Pregnancy test for women of child bearing potential

7.2 Weekly checklist during treatment:

- History and Physical
- Documentation of current medications
- Adverse effect assessment
- Hematology: CBC (hemoglobin, white blood cell count, platelets) with differential

7.3 Supportive Care

- Antiemetics will be ordered at the discretion of the attending physician.
- Mucositis/esophagitis may be ameliorated with sulcrafate or GI cocktail (e.g. Tylenol #3 suspension, bendaryl elixir, Maalox, viscous lidocaine).
- Intravenous hydration is recommended in patients with inadequate oral intake.
- Treatment-related diarrhea will be managed with high-dose loperamide. The recommended dose of loperamide is 4 mg initially (two capsules) then 2 mg after each loose stool, not to exceed 16 mg daily.

7.4 Follow-up checklist: Follow-up visits are to be done every 2 weeks for first 2 months;
 then every 3 months for 1 years; then every 6 months indefinitely

- History and Physical
- Documentation of current medications
- Adverse effect assessment
- CT scan of chest, abdomen, and pelvis (initially 4-6 weeks following completion of radiation therapy to assess response; then every 3 months for first year then every 6 months indefinitely)
- Bone scan (initially 4-6 weeks following completion of radiation therapy to assess response; then every 3 months for first year then every 6 months indefinitely). The bone scan is optional if the patient did not have any evidence of disease on the bone scan upon enrollment into the trial.
- Whole body 18-FDG PET/CT initially 3 months following completion of radiation therapy and every 3 months for the first year and every 6 months thereafter is an acceptable alternative to CT of chest, abdomen and pelvis and bone scan
- Plain X-ray and/or CT and/or MRI of bone abnormality seen on bone scan (if clinically indicated)
- MRI brain if clinically indicated
- Hematology: CBC (hemoglobin, white blood cell count, platelets) with differential
- Biochemistry: Comprehensive metabolic profile (glucose, sodium, potassium, chloride, carbon dioxide, BUN, creatinine, calcium, total bilirubin, alkaline phosphatase, AST, ALT)- (every 3 months for first 2 years then every 6 months indefinitely)
- Tumor markers, if elevated prior to treatment, are recommended
- 18-FDG PET scan every 6 months (optional).

**8.** **TREATMENT PLAN – EXTRACRANIAL STEREOTACTIC RADIOTHERAPY**

8.1 Radiotherapy Guidelines

1. All patients must meet radiotherapy inclusion criteria.
2. All patients will undergo CT simulation prior to the first treatment using the appropriate immobilization device**.** Examples are aquaplast mask for head and neck lesions and stereotactic body frame for thoracic and abdominal lesions.
3. All patients will undergo CT based treatment planning.
4. The definition of volumes will be in accordance with the 1993 ICRU Report #50: Prescribing, Recording and Reporting Photon Beam Therapy.
   1. A gross tumor volume (GTV) will be entered for each lesion, based on available imaging data.
   2. A clinical target volume (CTV) will be defined as the GTV plus a margin for microscopic extension.
   3. A planning target volume (PTV) will be determined which will correspond to CTV plus appropriate margin for tumor motion and set-up uncertainty. This generally will be between 3 and 10 mm.
5. The PTV will be treated to the prescribed dose.
6. Normal tissues must be contoured in order to determine dose volume histograms (DVH) include: both lungs (as the total lung volume) minus GTV, spinal cord, liver, kidney, heart, and esophagus.
7. The treatment plan used for each patient will be based on an analysis of the volumetric dose, including dose volume histogram of the PTV and normal structures. Either 3D “forward” planning or “inverse” planning with beamlet intensity modulated treatment planning is allowed.
8. Image guided treatment is mandatory. Method of image guidance will vary by site. Acceptable methods of image guidance include use of bone fusion, implanted fiducial markers or external optical tracking devices. For sites with possible respiratory motion, documentation of degree of tumor motion by using maximum inspiratory, expiratory and free breathing scans is mandatory. Use of an abdominal clamping device and real-time assessment of diaphragmatic motion by using external optical tracking is mandatory. Use of respiratory gating or active breathing control is optional.
9. Custom blocking will be used on all patients.
10. Treatment is to be delivered using a linear accelerator and photon energies ≥ 6 MV.
11. Radiation is to be delivered to each site over 10 fractions separated by at least 16 hours. Up to 5 sites may be treated. **(Added 7/27/06)** For patients with 3 to 5 sites of disease, it may be difficult to treat all sites of disease during one day. For these patients, an acceptable variant is to treat all 2 to 3 sites during days 8 to 19 and to treat the remaining sites on days 20 to 33. For these patients only, concurrent Sutent will continue until day 42.
12. Radiation Doses (Phase I): A phase I escalation of doses will be performed in order to determine in MTD in Gy

Doses of radiation will increase as follows in order to determine the MTD of radiation in combination with Sutent:

**Phase I Dose Levels**

| **Dose Level** | **Radiation Dose** | **Sutent Dose** |
| --- | --- | --- |
| 1 | 40 Gy in 10 fractions | 25 mg PO QD |
| 2 | 40 Gy in 10 fractions | 37.5 mg PO QD |
| 3 | 50 Gy in 10 fractions | 37.5 mg PO QD |
| 4 | 50 Gy in 10 fractions | 50 mg PO QD |
| 5 | 60 Gy in 10 fractions | 50 mg PO QD |

1. Radiation Doses (Phase II): The radiation dose used in the MTD dose level will be used throughout the phase II portion of this study.
2. Dose/volume constraints: The dose distribution will be converted to the normalized isoeffective dose, equivalent to dose given at standard fractionation (2 Gy per fraction). Normal tissue tolerance doses to be used as guidelines for treatment plan evaluation are: (based on the treatment on the sum of all involved sites):
3. Spinal cord:
   - 1. Must keep each dose < 300 cGy/fraction (therefore maximum total spinal cord dose will be 3000 cGy)
     2. Same segment of spinal cord must not be in more than 1 treatment field
   1. Skin: Maximum skin dose 40 Gy
   2. Lung: Volume receiving > 20 Gy less than 30%
   3. Liver: Volume receiving > 30 Gy less than 50%
   4. Kidney: Volume receiving > 20 Gy (both kidneys) should be less than 50%
   5. Esophagus: Maximum esophageal dose ≤ 40 Gy
   6. Heart: Volume >25 Gy less than 60% and maximum heart dose <30 Gy
   7. Major Vessels: Maximum dose 40 Gy
   8. Bone marrow: Must limit the radiation to < 30% of bone marrow

**9. DRUG THERAPY - SUTENT**

9.1 Phase I Dose (days 1-28):

| **Dose Level** | **Radiation Dose** | **Sutent Dose** |
| --- | --- | --- |
| 1 | 40 Gy in 10 fractions | 25 mg PO QD |
| 2 | 40 Gy in 10 fractions | 37.5 mg PO QD |
| 3 | 50 Gy in 10 fractions | 37.5 mg PO QD |
| 4 | 50 Gy in 10 fractions | 50 mg PO QD |
| 5 | 60 Gy in 10 fractions | 50 mg PO QD |

9.2 Phase II Dose (days 1-28): Phase II will be the MTD from Phase I

9.3 Phase I and II Maintenance dose: will be 50 mg PO QD

9.4 Schedule:

- One days 1-28, Sutent will be taken p.o. in the morning
- Two weeks after completion of any chemotherapy, maintenance Sutent in 6 week cycles (consisting of Sutent 50 mg PO QD weeks 1-4 followed by no treatment weeks 5-6) until progression or death

9.5 Formulation and Packaging: SU011248 is a small molecule inhibitor of the receptor tyrosine kinases VEGFR, PDGFR, KIT, and FLT3. In this clinical trial, study medication of SU011248 refers to the investigational agent SU011248 L-malate. Sunitinib L-malate salt will be supplied as hard gelatin capsules containing 12.5-mg, 25-mg, and 50-mg equivalents of Sunitinib malate free base, in bottles containing 30 capsules each."

9.6 Storage:Store at 25º C (*77º F*); excursions permitted to 15-30º C (*59-86º F*).

9.7 Adverse Effects: Data regarding the safety of Sutent are largely from a Phase III randomized trial of 304 patients with metastatic GI stromal tumors following imatinib failure who were treated with Sutent vs. placebo and 169 patients with metastatic renal cell carcinoma treated on phase II trails (32, 33).

Cardiac: Left Ventricular Dysfunction – The incidence of decreased left ventricular ejection fraction to below the lower limit of normal is 11-15% with Sutent vs. 3% for placebo. Over 40% of patients recover left ventricular ejection fraction without intervention. Only 1% of patients suffered grade 3 left ventricular systolic toxicity. Sutent should be discontinued in the presence of clinical congestive heart failure. The Sutent dose should be reduced in all patients without clinical congestive heart failure but with an ejection fraction of <50% and a drop of over 20% from baseline.

Hemorrhagic Events: Bleeding occurred in 18 to 26% of patients receiving Sutent vs. 17% for placebo with epistaxis being the most common hemorrhagic event. The incidence of Grade 3-4 bleeding is 1 to 7% among patients receiving Sutent vs. 9% for placebo. However, fatal pulmonary hemorrhage was noted in two patients with non-small cell lung cancer. Bleeding events, including fatal GI bleeding, has been noted in patients within 30 days of completing Sutent. Although there is no specific contraindication for concurrent anticoagulants, patients requiring continued Coumadin, lovenox, heparin, NSAIDs, aspirin, plavix or similar drugs will be excluded from this study (12/16/09).

Hypertension: Hypertension was noted in 15 to 28% of patients receiving Sutent vs. 11% for placebo. Grade 3 hypertension occurred in 4 to 6% of patients receiving Sutent vs. 0% for placebo. No case of grade 4 hypertension was reported. Careful monitoring and use of antihypertensive therapy is recommended. Sutent should be discontinued in cases of severe hypertension (defined as >200 mgHg systolic or >110 mgHg diastolic).

Adrenal: Adrenal toxicity was noted in rats and monkeys treated with Sutent but to date, there have been no cases of clinical adrenal insufficiency, adrenal necrosis or hemorrhage in human clinical trials. Nonetheless, patients receiving Sutent that experience stress such as surgery, trauma or severe infection should be monitored for adrenal insufficiency.

Gastrointestinal: Diarrhea, nausea, stomatitis, dyspepsia and vomiting were the most commonly reported gastrointestinal events occurring in patients who received Sutent. Diarrhea and stomatitis occurred more commonly in patients treated with Sutent than placebo. Supportive care for gastrointestinal adverse events, such as anti-nausea or anti-diarrheal medication should be prescribed as needed.

Skin: Skin discoloration, often to a yellowish hue occurred in approximately 1/3 of patients treated with Sutent. Depigmentation of the hair or skin, dryness, cracking, blistering or rash was also reported.

9.8 Potential Drug-Drug Interactions: SU11248 is mediated metabolized by the liver by CYP3A4. Therefore CYP3A4 inducers such as rifampin will result in a 56-79% reduction in SU11248 serum doses while CYP3A4 inhibitors such as ketoconazole will result in a 59-76% increase in SU11248 serum doses. For patients who are being concomitantly treated with a potent CYP3A4 inhibitor or CYP3A4 inducer, alternative treatments that lack potent CYP3A4-inducing properties should be considered. For patients who require treatment with a potent CYP3A4 inhibitor, a dose reduction should be considered in the presence of severe adverse events. For patients who require treatment with a potent CYP3A4 inducer, a dose increase should be considered.

However, in-vitro studies indicate that SU11248 does not induce or inhibit the major CYP450 enzymes including CYP3A4.

9.9 Administration: Sutent may be taken with or without food. The following schedule is recommended for the phase I and phase II studies:

| **REGISTER** | *Treatment* | *Days*  *1-7* | *8-12* | *13-14* | *15-19* | *20-28* | *29-42* |
| --- | --- | --- | --- | --- | --- | --- | --- |
|  | XRT |  | X |  | X |  |  |
|  | Sutent | X | X | X | X | X |  |

**Sutent will be taken by mouth every morning on days 1-28**

**Phase I Dose Levels**

| **Dose Level** | **Radiation Dose** | **Sutent Dose** |
| --- | --- | --- |
| 1 | 40 Gy in 10 fractions | 25 mg PO QD |
| 2 | 40 Gy in 10 fractions | 37.5 mg PO QD |
| 3 | 50 Gy in 10 fractions | 37.5 mg PO QD |
| 4 | 50 Gy in 10 fractions | 50 mg PO QD |
| 5 | 60 Gy in 10 fractions | 50 mg PO QD |

**Phase II dose level will be MTD determined by Phase I**

**↓**

Additional chemotherapy (as determined by Medical Oncology)

**↓**

Maintenance Sutent (until death or progression) and appropriate follow-up

- Two weeks after completion of any chemotherapy, maintenance Sutent in 6 week cycles (consisting of Sutent 50 mg PO QD weeks 1-4 followed by no treatment weeks 5-6) until progression or death

9.10 Dose Modifications for Sutent(Applies only to phase II)

Dose modification of Sutent can be secondary to any grade ≥3 toxicity related to Sutent according to the judgment of the treating physician. When toxicities eliciting dose modification occur, Sutent will be temporarily discontinued but restarted at the next lower dose level when the toxicity has resolved to grade 2 or lower. In the event of hospitalization for reasons other than drug toxicity, patients should be directed to continue trial drug at the discretion of their physician. The principal investigator should be notified immediately of any compliance issues that arise in the course of the trial. If a patient still experiences dose-modifying toxicities despite drug holiday and reduced dose schedule, the patient, if benefiting from treatment, should stop receiving Sutent and continue radiation.

The following toxicities, in addition to any other toxicity as judged by the treating physician, merit temporary discontinuation should be considered dose-limiting toxicity:

1. Any CTC grade 3 or 4 non-hematopoietic adverse event that the investigator considers consistent with a drug-related toxicity;

2. Any CTC grade 4 hematopoietic adverse event that the investigator considers consistent with a drug-related toxicity;

**10. PHASE I DOSE ESCALATION RULES**

- Both radiation dose and Sutent dose will be escalated in 5 dose levels.

**Table 1. Phase I Dose Levels**

| **Dose Level** | **Radiation Dose** | **Sutent Dose** |
| --- | --- | --- |
| 1 | 40 Gy in 10 fractions | 25 mg PO QD |
| 2 | 40 Gy in 10 fractions | 37.5 mg PO QD |
| 3 | 50 Gy in 10 fractions | 37.5 mg PO QD |
| 4 | 50 Gy in 10 fractions | 50 mg PO QD |
| 5 | 60 Gy in 10 fractions | 50 mg PO QD |

.

Phase I dose escalation will proceed according to a “3+3” design. After 3 patients have been treated on each dose level, they will be followed for at least 30 days. If 0 of 3 patients have DLTs, the dose will be escalated to the next dose level. If ≥2 of 3 patients have DLTs, dose escalation will be terminated and the next lowest dose level will be declared the maximal tolerated dose for Phase II evaluation. If 1 of 3 patients has a DLT, then three more patients will be treated at the same dose level and if the incidence of DLT among six patients is < 2 of 6, the dose will be escalated to the next level for that site.

- Grades 3-5 non-hematological toxicities (excluding nausea, vomiting, and alopecia) and Grade 4-5 hematological toxicities will be referred to as dose limiting toxicities (DLT).
  - Esophagitis and mucositis are very common side effects with radiation therapy and grade 3 side effects can be seen after palliative courses of radiotherapy. Therefore, grade 3 mucositis or esophagitis lasting ≤ 7 days will **not** be considered a DLT. If the grade 3 mucositis or esophagitis lasts > 7 days, it will then be considered a DLT.
- The recommended phase II dose will be 1 dose level below the defined MTD or at the maximum tested dose if the MTD has not been reached.

**11. ADDITIONAL INTERVENTIONS – CHEMOTHERAPY, SURGERY AND/OR ADDITIONAL RADIATION**

11.1 Interventions prior to protocol therapy: If chemotherapy, surgery and/or radiation are recommended by the treating physicians based on the patient’s clinical needs, they may be performed two weeks before or after days 1-28 of protocol therapy.

For instance, a patient with a history of breast cancer status post mastectomy (three years ago) and adjuvant chemotherapy now presents with a solitary brain metastasis, a single lung lesion and two bone lesions. The brain metastasis and lung lesion are both amenable to resection. Subsequently, the patient receives whole brain radiation and taxane based chemotherapy. After chemotherapy, CT of the chest abdomen and pelvis and MRI of the brain reveal no evidence of metastatic disease but bone scan demonstrates residual uptake in the two bone lesions. Two weeks after completion of chemotherapy, the patient is eligible for protocol therapy. The patient will receive Sutent on days 1-28 and extracranial stereotactic radiation on days 8-12 and 15-19 as per protocol. Two weeks after protocol therapy (day 43), the patient may receive additional chemotherapy. After completion of all planned chemotherapy, the patient will start maintenance Sutent. Therefore, all of the chemotherapy, surgery and whole brain radiation are allowed by protocol but are not considered “on protocol therapy”. While all toxicities of these interventions will be documented and recorded, any toxicity resulting from non-protocol therapy will not count as dose-limiting toxicity.

11.2 If patients have documented progression during additional chemotherapy and/or maintenance Sutent, the patient will be taken off protocol. At that time, any necessary intervention (chemotherapy, surgery and/or radiation will be allowed).

**12. TOXICITY**

- Radiation side effects are limited to the area involved in the treatment field(s). Acute and late toxicity related to radiation therapy include fatigue, nausea and vomiting, myelosuppression, skin erythema, subcutaneous fibrosis, esophagitis, pericarditis, diarrhea, cystitis, proctitis, myelitis, acute radiation pneumonitis, late pulmonary fibrosis, esophageal stricture, liver dysfunction, kidney damage, small bowel obstruction.
- Sutent toxicities are discussed in detail in Section 9.7
- Grade 3-5 non-hematological toxicities (excluding nausea, vomiting, and alopecia) and Grade 4-5 hematological toxicities will be referred to as dose limiting toxicities (DLT).
  - Esophagitis and mucositis are very common side effects with radiation therapy and grade 3 side effects can be seen after palliative courses of radiotherapy. Therefore, grade 3 mucositis or esophagitis lasting ≤ 7 days will not be considered a DLT. If the grade 3 mucositis or esophagitis lasts > 7 days, it will then be considered a DLT.
- All life-threatening Grade 4 and all fatal toxicities (Grade 5) must be reported immediately and evaluated.
- Acute toxicity: Acute side effects are considered as occurring ≤ 30 days from the start of radiation therapy. They will be documented using the NCI Common Terminology Criteria for Adverse Events (CTCAE) version 3.0 (A copy can be downloaded from [http://ctep.info.nih.gov](http://ctep.info.nih.gov/)).
- Late toxicity: Late side effects are considered occurring > 30 days from the start of radiation therapy. They will be evaluated and graded according to the RTOG Late Morbidity Scoring Scale (See Appendix).

**13. MEASUREMENT OF EFFECT**

- Response and progression will be evaluated in this study using the new international criteria proposed by the RECIST Committee. (38) Changes in only the largest diameter (unidimensional measurement) of the tumor lesions are used in the RECIST criteria.
- All lesions (except lesions seen on bone scan alone) are considered to be target lesions and are measurable. They are defined as being accurately measured in one dimension (longest diameter to be recorded). All tumors must be recorded in millimeters (or decimal fractions of centimeters) using a ruler or caliper.
- Lesions seen on bone scan only are not measurable and therefore are evaluated separately
- All baseline evaluation should be performed as closely as possible to beginning of the treatment and never more than 4 weeks before the beginning of treatment.
- The same method of assessment and the same technique should be used to characterize each identified and reported lesion at baseline and during follow-up.
- First assessment should be performed 4-6 weeks after completion of radiation therapy.
- Response Criteria for all lesions (except lesions seen only on bone scan):
  - Complete response (CR): Disappearance of all lesions
  - Partial response (PR): At least 30% decrease in the sum of the longest diameter (LD) of all lesions, taking as a reference the baseline sum LD
  - Progressive disease (PD): At least a 20% increase in the sum of the LD of the target lesions, taking as a reference the smallest sum LD recorded since the treatment started or the appearance of one or more new lesions
  - Stable disease (SD): Neither sufficient shrinkage to qualify for PR or sufficient increase to qualify for PD, taking as reference the smallest LD since the treatment started.
- Response Criteria for lesions seen only on bone scan:
  - Complete response (CR): Disappearance of lesion.
  - Stable disease (SD): Persistence of lesion on bone scan.
- Evaluation of best overall response:
  - The best overall response is the best response recorded from the start of the treatment until disease progression/recurrence (taking as reference for progressive disease the smallest measurements recorded since the treatment started). The patient’s best response assignment will depend on the achievement of both measurement and confirmation criteria:

**Table 3**

| **All Measurable Lesions** | **Lesions on bone scan only** | **New Lesions** | **Overall Response** |
| --- | --- | --- | --- |
| CR | CR | No | CR |
| CR | SD | No | PR |
| PR | SD | No | PR |
| SD | Any | No | SD |
| PD | Any | Yes or No | PD |
| Any | Any | Yes | PD |

CR = complete response; PD = progressive disease; PR = partial response; SD = stable disease

- Note:
  - Patients with a global deterioration of health status requiring discontinuation of treatment without objective evidence of disease progression at that time should be classified as having “symptomatic deterioration.” Every effort should be made to document the objective progression, even after discontinuation of treatment.
  - In some circumstances, it may be difficult to distinguish residual disease from normal tissue. When the evaluation of complete response depends on this determination, it is recommended that the residual lesion be investigated (fine needle aspirate/biopsy) before confirming the complete response status.
- Confirmatory Measurement/Duration of Response
  - Confirmation
    - To be assigned a status of PR or CR, changes in tumor measurements must be confirmed by repeat assessments that should be performed at least 16 weeks after the criteria for response are first met.
  - Duration of Overall Response
    - The duration of overall response is measured from the time measurement criteria are met for CR or PR (whichever is first recorded) until the first date that recurrent or progressive disease is objectively documented (taking as reference for progressive disease the smallest measurements recorded since the treatment started).
    - The duration of overall CR is measured from the time measurement criteria are first met for CR until the first date that recurrent disease is objectively documented
  - Duration of Stable Disease
    - Stable disease is measured from the start of the treatment until the criteria for progression are met, taking as reference the smallest measurements recorded since the treatment started.
- Progression-Free and Overall Survival
  - Progression-free (time to progression): From the date of registration to the date of progressive disease or death
  - Overall survival time: From the date of registration to the date of death or date of last patient contact if censored

**14. BIOLOGICAL CORRELATES**

Several recent studies demonstrated predictive value of circulating VEGF after treatment with radiation. High levels of VEGF prior to radiation and failure of VEGF to decrease after radiation have been correlated with poor outcomes after radiation. To determine if blood or urine VEGF levels predict for outcome in patients treated on this protocol, peripheral blood and urine will be collected prior to and after radiation and Sutent. For the pre-treatment and post-treatment blood collections, 19 ml of blood will be collected from each patient: two 5 ml red top tubes; one for serum extraction and one to go with the two 4.5 ml purple EDTA tubes for DNA analysis. Serum extraction will require a centrifuge, pipette and -70° C freezer for storage.

Serum and urine VEGF before and after radiation + Sutent will be measured by a commercially available ELISA kit and correlated with outcome (Oncogene Research). Additionally, the Mount Sinai Radiation Biology division has significant experience with screening for genetic polymophisms using denaturing high performance liquid chromatography and Surveyor nuclease assays (39). Therefore, we propose using this technology to analyze specific VEGFR polymorphisms from peripheral blood samples of treated patients to determine there is any correlation with outcome for any specific or class of genetic variants.

Serum/blood will be analyzed for the translational research component of this protocol and will be stored for future studies. If at any time the patient withdraws consent to store and use specimens, the material will be destroyed.

**15. ADVERSE EVENTS**

- - A **serious adverse event** (experience) or reaction is any untoward medical occurrence that at any dose: results in death, is life-threatening, requires inpatient hospitalization or prolongation of existing hospitalization, results in persistent or significant disability / incapacity, or is a congenital anomaly / birth defect.
  - The definition of serious adverse event (experience) also includes *important medical event*. Medical and scientific judgment should be exercised in deciding whether expedited reporting is appropriate in other situations, such as important medical events that may not be immediately life-threatening or result in death or hospitalization but may jeopardize the patient or may require intervention to prevent one of the other outcomes listed in the definition above. These should also usually be considered serious.
- Mount Sinai School of Medicine Reporting Guidelines
  - If the reaction requires reporting, the Research Nurse, MD or fellow reports adverse reaction to the patient data management office (212-241-7503) by the end of the business day when he/she becomes aware of the event. Events occurring after business hours will be reported to the patient data management office by 12 pm (noon) the next business day.
  - The following information is required when calling in the event:
    - Caller’s Name and Telephone Number, Patient Initials, Patient Medical Record Number, IRB Protocol Number, PI of Study, Attending Physician, Date of Event, Description of Event (including grade of the event and if the event required hospitalization)
  - E-mail is sent to the research nurse, attending physician and PI of the study informing them that adverse reaction notification has been received.
  - Required protocol documents must be sent to the data management office along with Mount Sinai’s IRB Adverse Event Form within **5 working days of event occurrence**. Once the forms are completed forward the original to the study PI. The PI will then review, sign and place folder in the QA Coordinator’s box. A weekly report of delinquent or pending documents will be forwarded to the principal investigator. All delinquent reporting (greater than 10 days from event occurrence) must include documentation of reason for delinquency and may require implementation of an action plan.
  - Once the appropriate AE documents have been received, the principal investigator forwards these to the IRB (Mount Sinai only), affiliate institutions if applicable. A copy will be forwarded to the appropriate Research Nurse.
- Data Safety and Monitoring
  - Data Safety and Monitoring will occur at the biweekly Mount Sinai School of Medicine, Department of Radiation Oncology phase I/II quality assurance meetings, which are lead by the department chairman (Richard Stock, M.D.) and include a medical oncologist (Scott Tagawa, M.D.) and patient advocate. At each meeting, all active studies will be reviewed for safety and progress toward completion. Toxicities and adverse events will be reviewed at each meeting and a Data Safety and Monitoring form will be completed for each protocol and signed by either the principal investigator, the chairman of the department or by his designate if the chairman is not available.

**15.1 Serious Adverse Event (SAE) Reporting to Pfizer**

Within 24 hours of first awareness of the event (immediately if the event is fatal or
life-threatening), Principal Investigator will report to the Pfizer by facsimile any Serious Adverse Event ("SAE," as defined below) that occurs during the SAE reporting period (as defined below) in a Study subject assigned to receive the Pfizer Product.  Principal Investigator will report such SAEs using an FDA MEDWATCH form and the Serious Adverse Event Fax Cover Sheet provided by Pfizer.  SAEs should be reported as soon as they are determined to meet the definition, even if complete information is not yet available. 

*SAE Definition:*  An SAE is any adverse event, without regard to causality, that is life-threatening or that results in any of the following outcomes: death; in-patient hospitalization or prolongation of existing hospitalization; persistent or significant disability or incapacity; or a congenital anomaly or birth defect.  Any other medical event that, in the medical judgment of the Principal Investigator, may
jeopardize the subject or may require medical or surgical intervention to prevent one of the outcomes listed above is also considered an SAE. A planned medical or surgical procedure is not, in itself, an SAE.  Also specifically excluded from this definition of SAE is any event judged by the Principal Investigator to represent progression of the malignancy under study, unless it results in death within the SAE Reporting Period.

*SAE Reporting Period:* The SAEs that are subject to this reporting provision are those that occur from after the first dose of the Pfizer Product through 28 days after discontinuation of the Pfizer Product. 

*Follow-Up Information:*  Institution will assist Pfizer in investigating any SAE and will provide any follow-up information reasonably requested by Pfizer.

*Regulatory Reporting:*  Reporting an SAE to Pfizer does not relieve
Institution of responsibility for reporting it to regulatory
authorities, as required.

**16. STATISTICAL CONSIDERATIONS**

- Phase I: The primary objectives are to determine the maximum tolerated dose (MTD) and dose limiting toxicity (DLT) of extracranial stereotactic radiation therapy in combination with Sutent when given to all sites of involved disease in patients with metastatic cancer.
- For dose escalation purposes, the major endpoint will be dose-limiting toxicity occurring within 30 days after the start of radiation therapy.
- Dose limiting toxicity is defined as follows: Grade 3-5 non-hematological toxicities (excluding nausea, vomiting, and alopecia) and Grade 4-5 hematological toxicities.
  - Esophagitis and mucositis are very common side effects with radiation therapy and grade 3 side effects can be seen after palliative courses of radiotherapy. Therefore, grade 3 mucositis or esophagitis lasting ≤ 7 days will not be considered a DLT. If the grade 3 mucositis or esophagitis lasts > 7 days, it will then be considered a DLT.
- The MTD for a specific area will be exceeded if 2 or more patients out of 6 (or a third or more) with disease at that site experience DLT at that dose. The dose immediately below will be recommended for further study.
- Phase II: *(Includes 3 to 6 patients from Phase I MTD):* The median progression free survival with optimal chemotherapy alone for the 4 most common tumor types (lung, breast, prostate and colorectal) ranges from 2.2 to 10.6 months, depending on primary tumor site and degree of pretreatment. Therefore, we will assume a median time to progression of 6.4 months (standard deviation 3.2 months) with chemotherapy alone. With 38 patients, this study has 87% power (α=0.05) to detect a 25% increase in time to progression. Also the 2 year freedom from progression is <5% with chemotherapy therapy and this study has 70% power (α=0.05) to demonstrate a 10% absolute increase in 2 year progression-free survival.
  - All patients included in the study will be assessed for response to treatment, even if there are major protocol treatment deviations or if they are ineligible (intent-to-treat population). Criteria described in Section 10 will be used to assign each patient to one of the following response categories: 1) complete response, 2) partial response, 3) stable disease, 4) progressive disease, 5) early death from malignant disease, 6) early death from toxicity, 7) early death because of other cause, or 8) unknown (not assessable, insufficient data).
  - All patients who have met the eligibility criteria will be included in the main analysis of the response rate. Patients in response categories 4 – 8 will be considered as failing to respond to treatment (disease progression).
  - All individual lesions will also be recorded as CR, PR, SD, or PD.
  - Overall survival is the time from the date of first study treatment until death from any cause.

Overall survival and progression-free survival (PFS) will be calculated using the Kaplan-Meier (1958) estimator.(40) Median survival times and their associated 90% confidence intervals will be derived using the method described in Brookmeyer and Crowley (1982).(41)

The percentage of patients with complete responses and the percentage with complete or partial responses will be calculated and confidence intervals derived. The pattern of failures (local or distant) will be summarized. Adverse effects will also be tabulated by type and level of severity. Potential prognostic variables, such as number of lesion sites at baseline, will be examined by fitting Cox (1972) regression models for progression-free and overall survival times.(42) Given the sample size, only univariate analysis will be performed.

- Accrual Rate and Study Duration
  - The estimated accrual will depend on toxicity encountered, but we anticipate enrolling 50-65 patients.
  - We anticipate enrolling 1-3 patients per month.

**17. CONFIDENTIALITY**

Study records that identify patients will be kept confidential. Study records will contain patients’ name, address, and medical history number and will be available to the study doctor, research nurse, and data coordinator. Data collected in this study will be maintained on a password protected computer that only the primary investigator, co-investigators, research nurse, and data coordinator will be able to access. Study records will be secured in locked offices in the department of radiation oncology. Neither patients’ name nor other personally identifying information will be used in any publication resulting from the research study.

**18. REFERENCES**

1. Kaplan E, Meier P. Nonparametric estimation from incomplete observations. J Am Stat Assoc 1958;58:457-81.

2. Bailar JC, 3rd, Gornik HL. Cancer undefeated. N Engl J Med 1997;336(22):1569-74.

3. DeVita VT, Hellman S, Rosenberg SA. Cancer: principles and practice of oncology. 7th ed. Philadelphia, PA; London: Lippincott Williams & Wilkins; 2005.

4. Fong Y, Cohen AM, Fortner JG, et al. Liver resection for colorectal metastases. J Clin Oncol 1997;15(3):938-46.

5. Billingsley KG, Burt ME, Jara E, et al. Pulmonary metastases from soft tissue sarcoma: analysis of patterns of diseases and postmetastasis survival. Ann Surg 1999;229(5):602-10; discussion 10-2.

6. Hellman S, Weichselbaum RR. Oligometastases. J Clin Oncol 1995;13(1):8-10.

7. Halsted W. The results of radical operations for the cure of cancer of the breast. Ann Surg 1907;46:1.

8. Keynes G. Carcinoma of the breast, the unorthodox view. Proc Cardiff M Soc 1954;40.

9. Fisher B. Laboratory and clinical research in breast cancer--a personal adventure: the David A. Karnofsky memorial lecture. Cancer Res 1980;40(11):3863-74.

10. Hellman S. Karnofsky Memorial Lecture. Natural history of small breast cancers. J Clin Oncol 1994;12(10):2229-34.

11. Shepherd FA, Rodrigues Pereira J, Ciuleanu T, et al. Erlotinib in previously treated non-small-cell lung cancer. N Engl J Med 2005;353(2):123-32.

12. Tannock IF, de Wit R, Berry WR, et al. Docetaxel plus prednisone or mitoxantrone plus prednisone for advanced prostate cancer. N Engl J Med 2004;351(15):1502-12.

13. Hurwitz H, Fehrenbacher L, Novotny W, et al. Bevacizumab plus irinotecan, fluorouracil, and leucovorin for metastatic colorectal cancer. N Engl J Med 2004;350(23):2335-42.

14. Slamon DJ, Leyland-Jones B, Shak S, et al. Use of chemotherapy plus a monoclonal antibody against HER2 for metastatic breast cancer that overexpresses HER2. N Engl J Med 2001;344(11):783-92.

15. Sandler AB, Gray R, Brahmer J, et al. Randomized phase II/III Trial of paclitaxel (P) plus carboplatin (C) with or without bevacizumab (NSC # 704865) in patients with advanced non-squamous non-small cell lung cancer (NSCLC): An Eastern Cooperative Oncology Group (ECOG) Trial - E4599. Proc Am Soc Clin Oncol 2005;23 (suppl)(16S):4a.

16. Gonzalez-Garcia I, Sole RV, Costa J. Metapopulation dynamics and spatial heterogeneity in cancer. Proc Natl Acad Sci U S A 2002;99(20):13085-9.

17. Tannock IF. Conventional cancer therapy: promise broken or promise delayed? Lancet 1998;351 Suppl 2:SII9-16.

18. Hanahan D, Weinberg RA. The hallmarks of cancer. Cell 2000;100(1):57-70.

19. Mehta N, Mauer AM, Hellman S, et al. Analysis of further disease progression in metastatic non-small cell lung cancer: implications for locoregional treatment. Int J Oncol 2004;25(6):1677-83.

20. Ragaz J, Olivotto IA, Spinelli JJ, et al. Locoregional radiation therapy in patients with high-risk breast cancer receiving adjuvant chemotherapy: 20-year results of the British Columbia randomized trial. J Natl Cancer Inst 2005;97(2):116-26.

21. Overgaard M, Hansen PS, Overgaard J, et al. Postoperative radiotherapy in high-risk premenopausal women with breast cancer who receive adjuvant chemotherapy. Danish Breast Cancer Cooperative Group 82b Trial. N Engl J Med 1997;337(14):949-55.

22. Kao J, Mundt AJ. Metastatic and Recurrent Tumors. In: Mundt AJ, Roeske JC, eds. Intensity Modulated Radiation Therapy: A Clinical Perspective. Hamilton: BC Decker; 2005:593-8.

23. Herfarth KK, Debus J, Lohr F, et al. Stereotactic single-dose radiation therapy of liver tumors: results of a phase I/II trial. J Clin Oncol 2001;19(1):164-70.

24. Timmerman RD, Forster KM, Chinsoo Cho L. Extracranial stereotactic radiation delivery. Semin Radiat Oncol 2005;15(3):202-7.

25. Nagata Y, Negoro Y, Aoki T, et al. Clinical outcomes of 3D conformal hypofractionated single high-dose radiotherapy for one or two lung tumors using a stereotactic body frame. Int J Radiat Oncol Biol Phys 2002;52(4):1041-6.

26. Gerszten PC, Ozhasoglu C, Burton SA, et al. CyberKnife frameless stereotactic radiosurgery for spinal lesions: clinical experience in 125 cases. Neurosurgery 2004;55(1):89-98; discussion -9.

27. Schefter TE, Kavanagh BD, Timmerman RD, Cardenes HR, Baron A, Gaspar LE. A phase I trial of stereotactic body radiation therapy (SBRT) for liver metastases. Int J Radiat Oncol Biol Phys 2005;62(5):1371-8.

28. Whyte RI, Crownover R, Murphy MJ, et al. Stereotactic radiosurgery for lung tumors: preliminary report of a phase I trial. Ann Thorac Surg 2003;75(4):1097-101.

29. Onishi H, Araki T, Shirato H, et al. Stereotactic hypofractionated high-dose irradiation for stage I nonsmall cell lung carcinoma: clinical outcomes in 245 subjects in a Japanese multiinstitutional study. Cancer 2004;101(7):1623-31.

30. Wersall P, Kavanagh BD. Stereotactic body radiation therapy for renal cell carcinoma. In: Kavanagh BD, Timmerman RD, eds. Stereotactic Body Radiation Therapy. New York: Lippincott Williams and Wilkins; 2005.

31. Koong AC, Le QT, Ho A, et al. Phase I study of stereotactic radiosurgery in patients with locally advanced pancreatic cancer. Int J Radiat Oncol Biol Phys 2004;58(4):1017-21.

32. Demetri GD, van Oosterom AT, Blackstein M, et al. Phase 3, multicenter, randomized, double-blind, placebo-controlled trial of SU11248 in patients (pts) following failure of imatinib for metastatic GIST. Proc Am Soc Clin Oncol 2005;23 (Suppl)(16S):4000a.

33. Motzer RJ, Rini BI, Michaelson MD, et al. Phase 2 trials of SU11248 show antitumor activity in second-line therapy for patients with metastatic renal cell carcinoma (RCC). Proc Am Soc Clin Oncol 2005;23 (Supp)(16S):4508a.

34. Faivre S, Delbaldo C, Vera K, et al. Safety, pharmacokinetic, and antitumor activity of SU11248, a novel oral multitarget tyrosine kinase inhibitor, in patients with cancer. J Clin Oncol 2006;24(1):25-35.

35. Schueneman AJ, Himmelfarb E, Geng L, et al. SU11248 maintenance therapy prevents tumor regrowth after fractionated irradiation of murine tumor models. Cancer Res 2003;63(14):4009-16.

36. Mauceri HJ, Hanna NN, Beckett MA, et al. Combined effects of angiostatin and ionizing radiation in antitumour therapy. Nature 1998;394(6690):287-91.

37. Crane CH, Ellis LM, Abbruzzese JL, al. e. Phase I trial evaluating the safety of bevacizumab with concurrent radiotherapy and capecitabine in locally advanced pancreatic cancer. J Clin Oncol 2006;24(7):1145-51.

38. Therasse P, Arbuck SG, Eisenhauer EA, et al. New guidelines to evaluate the response to treatment in solid tumors. European Organization for Research and Treatment of Cancer, National Cancer Institute of the United States, National Cancer Institute of Canada. J Natl Cancer Inst 2000;92(3):205-16.

39. Iannuzzi CM, Atencio DP, Green S, Stock RG, Rosenstein BS. ATM mutations in female breast cancer patients predict for an increase in radiation-induced late effects. Int J Radiat Oncol Biol Phys 2002;52(3):606-13.

40. Kaplan E, Meier P. Nonparametric estimation from incomplete observations. J Am Stat Assoc 1958;58:457-81.

41. Brookmeyer R, Crowley J. A confidence interval for the median survival time. Biometrics 1982;38:29-41.

42. Cox D. Regression models and life tables (with discussion). J Royal Stat Soc B 1972;34:187-202.

**APPENDIX I**

INFORMED CONSENT FORM

**APPENDIX II**PERFORMANCE STATUS SCALES

**ZUBROD PERFORMANCE SCALE**

0 Fully active, able to carry on all predisease activities without restriction *(Karnofsky 90-100)*.

1 Restricted in physically strenuous activity but ambulatory and able to carry out work of a light or sedentary nature. For example, light housework, office work *(Karnofsky 70-80)*.

2 Ambulatory and capable of all self-care but unable to carry out any work activities. Up and about more than 50% of waking hours *(Karnofsky 50-60)*.

3 Capable of only limited self-care, confined to bed or chair 50% or more of waking hours *(Karnofsky 30-40)*.

4 Completely disabled. Cannot carry on any self-care. Totally confined to bed or chair *(Karnofsky 10-20)*.

### **Appendix 3.** **Mount Sinai Department of Radiation Oncology Clinical Trials Data and Safety Monitoring Plan**

### **I. Clinical Trials Monitoring Committee**

The Clinical Trials Monitoring Committee is responsible for continued review and monitoring of all clinical trials conducted by the Mount Sinai Department of Radiation Oncology. This committee provides oversight of study progress and safety by review of accrual and adverse events at quarterly meetings. The Committee:

#### a) Reviews all clinical trials conducted at the Mount Sinai Department of Radiation Oncology for progress and safety.

#### b) Reviews all adverse events requiring expedited reporting as defined in the protocol.

#### c) Reviews reports generated by the Mount Sinai Department of Radiation Oncology data quality control review process (internal audit, quality assurance review, and response review) described in Section II of this document.

#### d) Submits recommendations for corrective actions to the Institutional Review Board.

#### e) Notifies the Study Chair of the recommended action.

#### f) Notifies external sites participating in multiple-institutional clinical trials coordinated by the Mount Sinai Department of Radiation Oncology of expedited adverse events and/or committee recommendations.

Members are appointed by the Director of the Mount Sinai Cancer Center Clinical Trials Office. Membership duration is flexible to maintain required depth and breadth of expertise related to the spectrum of clinical research conducted at the Cancer Center. Interim meetings are scheduled to address specific issues that require immediate attention to insure patient safety.

Membership

| Name | Discipline |
| --- | --- |
| George Raptis, M.D. | Medical Oncology |
| Richard G. Stock, M.D. | Radiation Oncology |
| Simon Hall, M.D. | Urology |
| Jamie A. Cesaretti, M.D., M.S. | Radiation Oncology |
| Scott Tagawa, M.D. | Medical Oncology |
| Ilene Willetts, Ph.D. | Patient Advocacy |
| Johnny Kao, M.D. | Radiation Oncology |

## A. Data and Safety Monitoring Plan

### 1. Requirements

All clinical trials conducted at the Cancer Center must have a satisfactory data and safety monitoring plan that is described in detail in the protocol. The Clinical Trials Monitoring Committee review insures patient safety and that the degree and frequency of data and safety monitoring for individual studies will be commensurate with the size, complexity and risks of the trial.

### 2. Elements of a Data and Safety Monitoring Plan

#### a) Delineation of oversight responsibilities (either external DSMB or Mount Sinai Department of Radiation Oncology Clinical Trials Monitoring Committee).

#### b) Description of data and safety review process.

#### c) Time table for submission of data, safety, and progress information to the DSMB or Clinical Trials Monitoring Committee, the IRB, and the sponsor.

#### d) Process to implement closure of studies when significant risks or benefits are identified.

#### e) Description of adverse event reporting procedures.

## B. Guidelines for Data and Safety Monitoring Implementation

### 1. Monitoring and Reporting Requirements

Mount Sinai Department of Radiation Oncology monitoring requirements for trials without an acceptable external DSMB are as follows:

#### a) Phase I Trials

#### Investigators will conduct continuous review of data and patient safety at their biweekly Clinical Trials Quality Assuarance meetings where the results of each patient’s treatment are discussed and the discussion is documented in the minutes. The discussion will include for each dose level: the number of patients, significant toxicities as described in the protocol, dose adjustments, and responses observed. Quarterly summaries will be submitted to the Clinical Trials Monitoring Committee for review.

#### b) Phase I/II and Phase II Trials

#### Data related to these trials are discussed at regularly scheduled Clinical Trials Quality Assuarance meetings where the results of each patient’s treatment are discussed and the discussion is documented in the minutes. The discussion will include for each treatment arm/dose level: the number of patients, significant toxicities as described in the protocol, dose adjustments, and responses observed. Twice yearly, summaries will be submitted to the Clinical Trials Monitoring Committee for review.

### 2. Review and Oversight Requirements

#### a) Adverse Event – Reported By Phone Within 24 Hours

Adverse events requiring expedited reporting by phone within 24 hours (as described in the protocol) will also be reported by phone to the Clinical Trials Monitoring Committee administrator within one business day. Confirmation that all appropriate parties were notified will be done at this time. Hardcopies or electronic versions of NCI ADEERS form (#3500) and/or any other documentation available at that time will also be reviewed by the Committee Chair who will determine if immediate action is required. Within 10 working days all subsequent SAE documentation that is available will be submitted with a completed Mount Sinai SAE Evaluation Checklist to the Committee Chair who will determine if further action is required. All information will be tracked in the Mount Sinai School of Medicine Department of Radiation Oncology database.

If the AE occurs on a multiple-institutional clinical trial coordinated by the Mount Sinai School of Medicine Department of Radiation Oncology, the Research Coordinator will insure that all participating sites are notified of the event and resulting action within one working day of the determination.

#### b) Adverse Event – Reported within 10 Days

Adverse events requiring expedited AE reports in writing within 10 working days (as described in the protocol) will be sent to the Mount Sinai School of Medicine Department of Radiation Oncology Research Coordinator. Hardcopies or electronic versions of NCI ADEERS form (#3500) or other required forms will be submitted along with a copy of the SAE Evaluation Checklist. The Committee Chair will review these forms and determine if further action is required. This information will be tracked in the Mount Sinai School of Medicine Department of Radiation Oncology database.

If the AE occurs on a multiple-institutional clinical trial coordinated by the Mount Sinai School of Medicine Department of Radiation Oncology, the Research Coordinator will insure that all participating sites are notified of the event and resulting action within one working day of the determination.

#### c) Study Progress – Quarterly Review

Study progress assessment to determine whether accrual projections are being met and to determine if the trial should be continued based upon the likelihood of timely completion are reviewed at quarterly Clinical Trials Monitoring Committee meetings. Cumulative reports of adverse events requiring expedited reporting and any new adverse event requiring expedited reporting are also reviewed at the committee’s quarterly meetings.

An overall assessment of accrual, toxicities as described in the protocol, and responses will enable the committee members to assess whether significant benefits or risks are occurring that would warrant study closure. This information is provided by meeting minutes, internal audit and/or response review reports. The committee may request external DSMB reports or further information from the Study Chair.

The Clinical Trials Monitoring Committee recommendations for modifications to the trial are forwarded to the Institutional Review Board. The Study Chair is notified of this recommendation in order that he/she may alert all investigators, at the Mount Sinai School of Medicine Department of Radiation Oncology and at external sites involved in the trial, about the potential action. At this time the Study Chair may submit to the Institutional Review Board additional information that could affect the Committee’s decision. The Institutional Review Board will notify the Study Chair if they concur with the Clinical Trials Monitoring Committee recommendations, including suspension or closure. The Study Chair will notify all investigators involved with the study at the Mount Sinai School of Medicine Department of Radiation Oncology and external sites, the IRB, the sponsor and the funding agency and provide written documentation of these notifications to the Institutional Review Board.

#### d) Review of Adverse Event Rates

Once a month, adverse event rates will be monitored utilizing the Mount Sinai School of Medicine Department of Radiation Oncology Clinical Trials database. If any study has had two or more of the same AE reported in a month or more than six of the same AE in six months, the CTMC Chair will review the summary of SAEs, discuss events with Study Chair, and conduct a more detailed review with the Study Chair or the external DSMB if warranted. The Committee Chair will determine if further action is required. If this occurs on a multiple-institutional clinical trial coordinated by the Mount Sinai School of Medicine Department of Radiation Oncology, the principal investigator will insure that all participating sites are notified of the resulting action.

# II. Data Quality Control

Three types of procedures insure that Mount Sinai School of Medicine Department of Radiation Oncology protocol/patient data are of the highest quality. These are auditing, response review, and quality assurance review. Reports of these quality control activities are submitted and discussed at the quarterly Clinical Trials Monitoring Committee meeting and then submitted with recommendations for follow-up or corrective action to the Institutional Review Board. The Institutional Review Board reviews the reports, implements corrective action, and notifies the Clinical Research Committee of these actions. This review process is detailed in Mount Sinai School of Medicine Department of Radiation Oncology quality control standard operating procedures.

## A. Internal Audit

The audit procedure is a formal, comprehensive, source document review of any institutional study not otherwise audited by an external agency. Twice a year, two trials from this group are selected for audit. Ten percent of the cases from these trials are randomly selected for review. Typically two members of the clinical research staff and one clinical investigator, who is not a principal in the study, serve as auditors. The following elements are reviewed:

### 1. Source document verification of eligibility, response, and toxicity.

### 2. Regulatory review of IRB compliance and external reporting requirements.

### 3. Drug accountability and handling.

### 4. Completeness and quality of data.

## B. Response Review

Response Review is performed prior to quarterly Clinical Trials Monitoring Committee meetings on all cases exhibiting a partial response or complete response on any therapeutic clinical trial conducted at the Mount Sinai School of Medicine Department of Radiation Oncology. An independent investigator who is not a principal involved in the study, reviews and confirms measurements and source documentation.

## C. Quality Assurance Review

Quality Assurance Review is performed on all therapeutic clinical trials conducted at the Mount Sinia School of Medicine. The statistician randomly selects five cases for Quality Assurance Review every quarter from all studies at risk for review in this group. Typically two members of the clinical research staff, not involved in the conduct of the study, serve as reviewers. The Quality Assurance Review concentrates on data management and system procedures, quality of data collection and protocol adherence. The following elements are reviewed.

### 1. Eligibility and treatment compliance.

### 2. Assessment of disease status.

### 3. Completeness and quality of data.

### 4. Compliance with reporting procedures (including adverse events requiring expedited reporting).

### 5. Research chart organization.

# III. Conflict of Interest

The Clinical Affairs Committee is responsible for identifying, during their scientific review, potential conflicts of interest involved in any Mount Sinai School of Medicine Department of Radiation Oncology clinical trial. Investigators must indicate on the protocol submission form any potential conflict of interest resulting from their involvement in the trial. If a potential conflict is identified by the Committee, the investigator must work with his/her department chair and the Mount Sinai Conflict of Interest Committee to create a plan to eliminate or manage the conflict of interest. The Mount Sinai School of Medicine Policies and Procedures for Conflict of Interest govern this process.

**Oversight And Monitoring Plan**

The MSSM Clinical Trials Monitoring Committee (CTMC) is responsible for monitoring data quality and patient safety for all Mount Sinai School of Medicine Department of Radiation Oncology clinical studies. A summary of CTMC activities follows:

● Review of all clinical trials conducted at the Mount Sinai Department of Radiation Oncology for progress and safety

1. ● Review of all adverse events requiring expedited reporting as defined in the protocol
2. ● Review of reports generated by the Mount Sinai Department of Radiation Oncology data quality control review process
3. ● Submit recommendations for corrective action to the Clinical Affairs Committee (CAC)
4. ● Notify the Study Chair of the CTMC recommendation to the CAC
5. ● Notify external sites participating in multiple-institutional clinical trials coordinated by the Mount Sinai Department of Radiation Oncology of adverse events requiring expedited reporting and subsequent committee recommendations for study modifications.

**Monitoring And Reporting Guidelines**

**Phase I Trials**

Investigators will conduct continuous review of data and patient safety at their weekly Phase I review meetings where the results of each patient’s treatment are discussed and the discussion is documented in the minutes. The discussion will include for each dose level: the number of patients, significant toxicities as described in the protocol, doses adjustments, and responses observed. Quarterly summaries will be submitted to the Clinical Trials Monitoring Committee for review.

**Phase I/II and Phase II Trials**

Data related to these trials are discussed at regularly scheduled Phase I review meetings where the result of each patient’s treatment is discussed and the discussion is documented in the minutes. The discussion will include for each treatment arm/dose level, the number of patients, significant toxicities as described in the protocol, dose adjustments, and responses observed. Twice yearly, summaries will be submitted to the Clinical Trials Monitoring Committee for review.

POLICY & PROCEDURES FOR DATA AND SAFETY MONITORING

REVIEW AND OVERSIGHT REQUIREMENTS

1. a) Adverse Event – Reported By Phone Within 24 Hours

Adverse events requiring expedited reporting by phone within 24 hours (as described in the protocol) will also be reported by phone to the Clinical Trials Monitoring Committee administrator within one working day. Confirmation that all appropriate parties were notified will be done at this time. Hardcopies or electronic versions of NCI ADEERS form (#3500) and/or any other documentation available at that time will also be reviewed by the Committee Chair who will determine if immediate action is required. Within ten working days all subsequent SAE documentation that is available will be submitted with a completed Mount Sinai Department of Radiation Oncology SAE Evaluation Checklist to the Committee Chair who will determine if further action is required. All information will be tracked in the Mount Sinai Department of Radiation Oncology database.

If the AE occurs on a multiple-institutional clinical trial coordinated by the Mount Sinai Department of Radiation Oncology, the Research Coordinator will insure that all participating sites are notified of the event and resulting action within one working day of the determination.

1. b) Adverse Event – Reported within 10 Days

Adverse events requiring expedited AE reports in writing within 10 working days (as described in the protocol) will be sent to the Mount Sinai Department of Radiation Oncology Researc Coordinator. Hardcopies or electronic versions of NCI ADEERS form (#3500) or other required forms will be submitted along with a copy of the SAE Evaluation Checklist. The Committee Chair will review these forms and determine if further action is required. This information will be tracked in the Mount Sinai Department of Radiation Oncology database.

If the AE occurs on a multiple-institutional clinical trial coordinated by the Mount Sinai Department of Radiation Oncology, the Research Coordinator will insure that all participating sites are notified of the event and resulting action within one working day of the determination.

1. c) Study Progress – Quarterly Review

Study progress assessment to determine whether accrual projections are being met and to determine if the trial should be continued based upon the likelihood of timely completion are reviewed at quarterly Clinical Trials Monitoring Committee meetings. Cumulative reports of adverse events requiring expedited reporting and any new adverse event requiring expedited reporting are also reviewed at the committee’s quarterly meetings.

An overall assessment of accrual, toxicities as described in the protocol, and responses will enable the committee members to assess whether significant benefits or risks are occurring that would warrant study closure. This information is provided by meeting minutes, internal audit and/or response review reports. The committee may request external DSMB reports or further information from the Study Chair.

POLICY & PROCEDURES FOR DATA AND SAFETY MONITORING

The Clinical Trials Monitoring Committee recommendations for modifications to the trial are forwarded to the Institutional Review Board. The Study Chair is notified of this recommendation in order that he/she may alert all investigators, at the Mount Sinai Department of Radiation Oncology and at external sites involved in the trial, about the potential action. At this time the Study Chair may submit to the Clinical Affairs Committee additional information that could affect the Committee’s decision. The Clinical Affairs Committee will notify the Study Chair if they concur with the Clinical Trials Monitoring Committee recommendations, including suspension or closure. The Study Chair will notify all investigators involved with the study at Mount Sinai Department of Radiation Oncology and external sites, the IRB, the sponsor and the funding agency and provide written documentation of these notifications to the Clinical Affairs Committee.

d) Review of Adverse Event Rates

Once a month, adverse event rates will be monitored utilizing the Mount Sinai Department of Radiation Oncology Clinical Trials database. If any study has had two or more of the same AE reported in a month or more than six of the same AE in six months, the CTMC Chair will review the summary of SAEs, discuss events with Study Chair, and conduct a more detailed review with the Study Chair or the external DSMB if warranted. The Committee Chair will determine if further action is required.

If this occurs on a multiple-institutional clinical trial coordinated by the Mount Sinai Department of Radiation Oncology, the Safety Coordinator will insure that all participating sites are notified of the resulting action.

EXPEDITED REPORTING OF ADVERSE EVENTS

Depending on the nature, severity, and attribution of the event an ADR report will be phoned in, submitted in writing, or both according to Tables below. Telephoned Adverse Events must also be reported by phone to the Mount Sinai Department of Radiation Oncology Clinical Trials Monitoring Committee within one working day of the event. All adverse events must also be reported to the Mount Sinai IRB, and any sponsor/funding agency not already included in the list.

| Telephone reports to:  (Investigator: Insert names and phone numbers for required notifications) •   1. Mount Sinai Clinical Trials Monitoring Committee Administrator 212-241-8617- within one working day of the event |
| --- |
| Written reports to:  (Investigator: Insert names, fax numbers, and addresses for required notifications)   1. Mount Clinical Trials Monitoring Committee Administrator –   FAX 212-289-2663 or deliver to Clinical Trials Office, Division of Hematology Oncology at Atran 2nd Floor   1. Mount Sinai IRB – Copy of final written report to Sponsor. |

| TABLE 1 | | | |
| --- | --- | --- | --- |
| Summary Of Reporting Requirements For Adverse Events On Trials Where The Investigator Holds IND | | | |
| EXPEDITED REPORTING FOR PHASE I STUDIES | | | |
| Unexpected Event | | Expected Event | |
| GRADES 2 - 3  Attribution of Possible, Probable, or Definite | GRADES 4 and 5  Regardless of Attribution | GRADES 1 - 3 | GRADES 4 and 5,  Regardless of Attribution |
| Grade 2 - Expedited report within 15 working days to FDA.  Grade 3 - Report by phone to FDA within 24 hrs. Expedited report to follow within 15 working days.  (Grade 1 - Adverse Event Expedited Reporting NOT required.) | Report by phone to FDA within 24 hrs. Expedited report to follow within 15 working days.  This includes all deaths within 30 days of the last dose of treatment with an investigational agent regardless of attribution.  Any late death attributed to the agent (possible, probable, or definite) should be reported within 15 working days. | Adverse Event Expedited Reporting NOT required. | Report by phone to FDA within 24 hrs. Expedited report to follow within 15 working days.  This includes all deaths within 30 days of the last dose of treatment with an investigational agent regardless of attribution.  Any late death attributed to the agent (possible, probable, or definite) should be reported within 15 working days. |

For Hospitalization Only – Any medical event equivalent to the CTC Grade 3,4,5 which precipitated hospitalization (or prolongation of existing hospitalization) must be reported regardless of requirements for phase of study, expected or unexpected and attribution.

Expedited reporting may not be appropriate for specific expected adverse events for certain later phase 2 and phase 3 protocols. In those situations the adverse events that will not have expedited reporting must be specified in the text of the approved protocol. An expected Grade 3 event that is using the generic reporting criteria, for instance. In a trial of investigational agents where grade 3 diarrhea requiring hospitalization is expected, only diarrhea requiring ICU care (Grade 4) might be designated for expedited reporting.

| TABLE 2 | | | |
| --- | --- | --- | --- |
| Summary Of Reporting Requirements For Adverse Events On Trials Where The Investigator Holds IND | | | |
| EXPEDITED REPORTING FOR PHASE 2 AND PHASE 3 STUDIES | | | |
| Unexpected Event | | Expected Event | |
| GRADES 2 - 3  Attribution of Possible, Probable, or Definite | GRADES 4 and 5  Regardless of Attribution | GRADES 1- 3 | GRADES 4 and 5,  Regardless of Attribution |
| Expedited report within 15 working days to FDA.  (Grade 1 - Adverse Event Expedited Reporting NOT required.) | Report by phone to FDA within 24 hrs. Expedited report to follow within 15 working days.  This includes all deaths within 30 days of the last dose of treatment with an investigational agent regardless of attribution.  Any late death attributed to the agent (possible, probable, or definite) should be reported within 15 working days. | Adverse Event Expedited Reporting NOT required. | Report by phone to FDA within 24 hrs. Expedited report to follow within 15 working days.  This includes all deaths within 30 days of the last dose of treatment with an investigational agent regardless of attribution.  Any late death attributed to the agent (possible, probable, or definite) should be reported within 15 working days.  Grade 4 Myelosuppression or other Grade 4 events that do not require expedited reporting will be specified in the protocol. |

For Hospitalization Only – Any medical event equivalent to the CTC Grade 3,4,5 which precipitated hospitalization (or prolongation of existing hospitalization) must be reported regardless of requirements for phase of study, expected or unexpected and attribution.

Expedited reporting may not be appropriate for specific expected adverse events for certain later phase 2 and phase 3 protocols. In those situations the adverse events that will not have expedited reporting must be specified in the text of the approved protocol. An expected Grade 3 event that is using the generic reporting criteria, for instance. In a trial of investigational agents where grade 3 diarrhea requiring hospitalization is expected, only diarrhea requiring ICU care (Grade 4) might be designated for expedited reporting.

| TABLE 3 | | | |
| --- | --- | --- | --- |
| Summary Of Reporting Requirements For Adverse Events On TrialsInvolving Commercial Agents With No IND Is Voluntary (Med Watch Form) | | | |
| EXPEDITED REPORTING FOR PHASE I STUDIES | | | |
| Unexpected Event | | Expected Event | |
| GRADES 2 - 3  Attribution of Possible, Probable, or Definite | GRADES 4 and 5  Regardless of Attribution | GRADES 1-3 | GRADES 4 and 5,  Regardless of Attribution |
| Grade 2 - Expedited report within 15 working days to FDA.  Grade 3 - Report by phone to FDA within 24 hrs. Expedited report to follow within 15 working days.  (Grade 1 - Adverse Event Expedited Reporting NOT required.) | Report by phone to FDA within 24 hrs. Expedited report to follow within 15 working days.  This includes all deaths within 30 days of the last dose of treatment with an investigational agent regardless of attribution.  Any late death attributed to the agent (possible, probable, or definite) should be reported within 15 working days. | Adverse Event Expedited Reporting NOT required. | Report by phone to FDA within 24 hrs. Expedited report to follow within 15 working days.  This includes all deaths within 30 days of the last dose of treatment with an investigational agent regardless of attribution.  Any late death attributed to the agent (possible, probable, or definite) should be reported within 15 working days. |

NOTE: Use Med Watch Form

For Hospitalization Only – Any medical event equivalent to the CTC Grade 3,4,5 which precipitated hospitalization (or prolongation of existing hospitalization) must be reported regardless of requirements for phase of study, expected or unexpected and attribution.

Expedited reporting may not be appropriate for specific expected adverse events for certain later phase 2 and phase 3 protocols. In those situations the adverse events that will not have expedited reporting must be specified in the text of the approved protocol. An expected Grade 3 event that is using the generic reporting criteria, for instance. In a trial of investigational agents where grade 3 diarrhea requiring hospitalization is expected, only diarrhea requiring ICU care (Grade 4) might be designated for expedited reporting.

| TABLE 4 | | | |
| --- | --- | --- | --- |
| Summary Of Reporting Requirements For Adverse Events On TrialsInvolving Commercial Agents With No IND Is Voluntary (Med Watch Form) | | | |
| EXPEDITED REPORTING FOR PHASE 2 AND PHASE 3 STUDIES | | | |
| Unexpected Event | | Expected Event | |
| GRADES 2 - 3  Attribution of Possible, Probable, or Definite | GRADES 4 and 5  Regardless of Attribution | GRADES 1 -3 | GRADES 4 and 5,  Regardless of Attribution |
| Expedited report within 15 working days to FDA.  (Grade 1 - Adverse Event Expedited Reporting NOT required.) | Report by phone to FDA within 24 hrs. Expedited report to follow within 15 working days.  This includes all deaths within 30 days of the last dose of treatment with an investigational agent regardless of attribution.  Any late death attributed to the agent (possible, probable, or definite) should be reported within 15 working days. | Adverse Event Expedited Reporting NOT required. | Report by phone to FDA within 24 hrs. Expedited report to follow within 15 working days.  This includes all deaths within 30 days of the last dose of treatment with an investigational agent regardless of attribution.  Any late death attributed to the agent (possible, probable, or definite) should be reported within 15 working days.  Grade 4 Myelosuppression or other Grade 4 events that do not require expedited reporting will be specified in the protocol. |

Note: Use Med Watch Form

For Hospitalization Only – Any medical event equivalent to the CTC Grade 3,4,5 which precipitated hospitalization (or prolongation of existing hospitalization) must be reported regardless of requirements for phase of study, expected or unexpected and attribution.

Expedited reporting may not be appropriate for specific expected adverse events for certain later phase 2 and phase 3 protocols. In those situations the adverse events that will not have expedited reporting must be specified in the text of the approved protocol. An expected Grade 3 event that is using the generic reporting criteria, for instance. In a trial of investigational agents where grade 3 diarrhea requiring hospitalization is expected, only diarrhea requiring ICU care (Grade 4) might be designated for expedited reporting.

**Standard Operating Procedure for Blood and Urine Collection and Storage for Study 06-0906**

*Effective: 10/12/2006*

**Objective**

The objective of this procedure is to standardize the method for collecting venous blood specimens obtained by standard phlebotomy procedures and urine for storage.

**1. Summary of Method – Venous Blood Collection**

Venous blood is obtained by any standard phlebotomy technique from a peripheral access point, or from a central line into a Vacutainer tube.

**2. Summary of Method – Urine Collection**

Mid-stream urine is obtained by standard urine collection techniques into a sterile container.

**3) Maintaining the integrity of the samples and the confidentiality of subjects**

The specimens are stored in ampoules in liquid nitrogen canisters located in the locked laboratory of Dr. Barry S. Rosenstein and Johnny Kao in room Atran 206. Only the code for each patient is located on the ampoule. The link for the patients identify with the code is kept in a locked cabinet in a separate room.

**4) Future Use of Stored Specimens**

The samples will be used in the future if the PI of the project determines they will be useful for additional research. The expectation is to preserve the right for unlimited future use. The PI of the project, Dr. Johnny Kao, will decide the future use.

**5) Length of Storage**

The samples will be maintained indefinitely or until the patient withdraws consent.

**6) At what point will it be impossible for a subject to withdraw from the study and insist on the samples destruction.**

It will always be possible for a subject to withdraw from the study and insist on their sample’s destruction.
